# Weak Radiofrequency Field Effects on Biological Systems Mediated through the Radical Pair Mechanism

**DOI:** 10.1021/acs.chemrev.5c00178

**Published:** 2025-07-14

**Authors:** Luca Gerhards, Andreas Deser, Daniel R. Kattnig, Jörg Matysik, Ilia A. Solov’yov

**Affiliations:** † Institute of Physics, 11233 Carl von Ossietzky Universität Oldenburg, Carl-von-Ossietzky Str. 9-11, 26129 Oldenburg, Germany; ‡ Competence Center for Electromagnetic Fields, 39419 Federal Office for Radiation Protection, Karl-Liebknecht-Straße 33, 03046 Cottbus, Germany; § Living Systems Institute, 3286 University of Exeter, Stocker Road, Exeter, Devon EX4 4QD, United Kingdom; ∥ Department of Physics, 3286 University of Exeter, Stocker Road, Exeter, Devon EX4 4QL, United Kingdom; ⊥ Institut für Analytische Chemie, 9180 Universität Leipzig, Linnéstr. 3, 04103 Leipzig, Germany; # Research Center for Neurosensory, Science, 11233 Carl von Ossietzky Universität Oldenburg, 26111 Oldenburg, Germany; ∇ Center for Nanoscale Dynamics (CENAD), Institute of Physics, Carl von Ossietzky Universität Oldenburg, Ammerländer Heerstr. 114-118, 26129 Oldenburg, Germany

## Abstract

The widespread use of radiofrequency (RF) communication has increased the exposure of organisms to electromagnetic fields, sparking a debate over the potential health effects of weak RF electromagnetic fields. While some experimental studies suggest that low-amplitude RF radiation may influence cellular metabolism or sleep patterns or even promote cancer, these claims remain controversial due to limited theoretical plausibility. Central to this debate is the radical pair mechanism (RPM), a quantum-mechanical framework proposed to mediate RF effects. Despite its role in magnetoreception and various magnetic field effects on chemical reactions, the RPM often fails to align with observations at low, nonthermal RF field strengths. This review examines these contrasting perspectives by discussing experimental findings and theoretical models that aim to explain putative biological effects of RF magnetic fields. Emphasis is placed on the challenges of reconciling theoretical predictions with empirical data, particularly in the context of weak RF exposure. Additionally, an overview of the theoretical framework used in current modeling efforts highlights the complexity of applying the RPM to biological systems and underscores the importance of critical interpretation. The goal is to clarify the state of understanding and inform future research on RPM-mediated biological effects under weak RF exposure.

## Graphical abstract

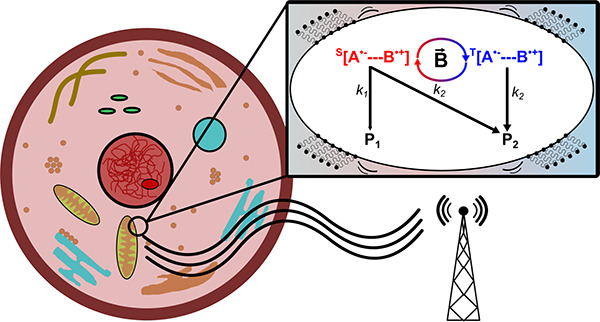

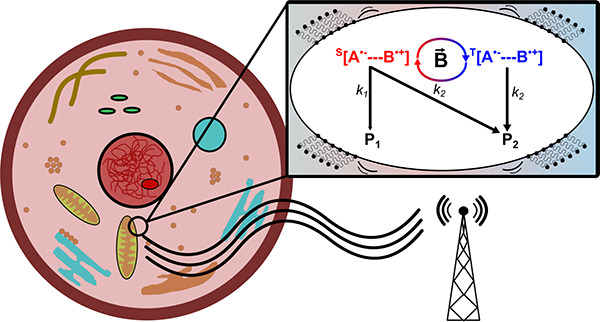

## 1 Introduction

Radiofrequency (RF) communication technologies are ubiquitous in modern society and result in an almost continuous exposure of residential areas to electromagnetic fields (EMF).^1–3^ RF-EMF also occurs naturally due to lightning and extraterrestrial processes and, as such, has always been present.^2,4^ However, the rapid increase of sources of *anthropogenic* RF-EMFs has raised public concerns about the possible adverse effects of permanent exposure to RF-EMFs.^1,3^

Motivated by the potential increase in exposure to RF-EMFs, several studies claimed that RF-EMFs could be harmful to the environment and especially to human health.^1,5,6^ For example, public concerns grew during the introduction of the 5G mobile phone network, leading to the formation of anti-5G groups, petitions to governments, and protests throughout the globe.^1,7^

The debate is also fueled by a plethora of experimental and epidemiological studies that claim that RF-EMF exposure has an impact on various biological systems.^8–15^ Among the health endpoints investigated are the alteration of cellular metabolism,^13,16,17^ the disruption of sleep,^18^ and cancer promotion.^19,20^ Despite occasional criticism of selected studies due to the lack of reproducibility^21^ and other issues, such as the isolation of an experimental setup against external field contamination when studying low amplitude (<1.2 μT) RF-EMF regimes,^22^ more and more studies appear claiming that biological effects arise under the influence of exposure to weak RF-EMF.^23–25^

However, the precise mechanism of how weak RF-EMFs might influence biological systems is still unknown. Although RF-EMFs were classified as possibly carcinogenic by the International Agency for Research on Cancer,^26,27^ such an association has not been scientifically established, and results of recent epidemiological studies do not indicate a higher cancer risk. The World Health Organization (WHO) initiated systematic reviews and meta-analyses to clarify indications about possible effects for several health endpoints, including cancer, adverse reproductive outcomes, cognitive impairment, and oxidative stress.^26,28–41^ The WHO has also planned to publish a monograph on radiofrequency fields and health that contains a risk assessment.^42^ A precise biophysical mechanism of how nonionizing and nonthermal RF-EMFs might influence biological systems relevant to any health endpoints remains elusive at the molecular level.

Motivated to understand the potential effect of RF-EMF exposure on biological systems, several theoretical models arose within the context of magnetobiology. However, most of these theories reveal inconsistencies and are not suitable for a comprehensive explanation of the mechanisms of biological action.^12^ By far, the most prominent and promising candidate for explaining the possible effects of weak RF-EMF is the radical pair mechanism (RPM).^43–46^

The RPM appears in the context of spin-correlated radical pairs (SCRP), which interact within an external MF, leading to an altered interconversion between the spin quantum states of the SCRP. Spin-correlated radical pairs are short-lived reaction intermediates that are usually formed in nonequilibrium electron spin states.^47^ When different spin states can undergo spin-selective reaction pathways, external magnetic fields (MF) can change the ratio between spin-selective reaction pathways, even though the magnetic interaction energies are a million times smaller than the thermal energy *k*_B_ *T* for a biological system at body temperature.

The RPM is a well-known mechanism that was already intensively studied in the context of spin chemistry.^43,48–53^ Furthermore, the RPM has gained even more attention as one of the leading theories to explain magnetoreception in many animal species capable of sensing the geomagnetic field (∼50 μT).^43,54,55^ In the context of magnetoreception, in behavioral experiments it was observed that extremely weak (on the order of pT/Hz^1/2^ for broadband exposure) RF-EMF exposure disrupts the navigational abilities of songbirds, leading to their disorientation.^56–59^ Other animals, such as the German cockroach (*Blatella germanica*)^60^ or the antarctic amphipod (*Gondogeneia antarctica*),^61^ were also found to be affected by exposure to RF-MF.

Hence, the RPM is frequently employed as a potential mechanism in studying magnetic field effects in many other biological systems^9,62–65^ and is, moreover, hypothesized as a possible cause for biologically relevant RF-EMF effects observed in several experimental studies.^66,67^ For example, the RPM is frequently associated with the increased formation of reactive oxygen species (ROS) within biological systems (e.g., related to the metabolism of nicotinamide adenine dinucleotide phosphate (NADPH) oxidase,^68^ a lipid protein involved in the cellular defense mechanism via the formation of superoxide radicals) under the influence of external magnetic fields.^69–72^

Although several experimental findings correlate the RPM with observed results, many questions remain open in relation to the effects of low-amplitude (<μT regime) RF-EMFs on an SCRP, as revealed through theoretical studies.^47,73^ Although it was demonstrated that at higher flux densities (≥μT regime) magnetic field effects (MFE) can be observed through applied RF-EMFs,^74^ at very weak EMFs (≪50 μT) theoretical calculations do not reveal pronounced effects unless the coherence lifetime of the SCRP is assumed to exceed several microseconds, which appears unrealistic in most scenarios.^75^ Furthermore, other effects, such as spin relaxation processes,^73,76,77^ which are dominant aspects when investigating SCRPs in biological environments, also counteract the possible interaction of an SCRP with a weak external MF. Theoretical results, based on current models, do not provide a quantitatively consistent explanation of the possibility of RF-EMF effects at low amplitudes and fuel the criticism of experimental studies^73^ claiming that weak RF-MFs have an impact on biological systems or even on human health.^78^

The discrepancy between theoretical calculations and experimental observations demonstrates a knowledge gap that requires additional research. This review provides an overview of the current controversy in experimental studies and the current state-of-the-art theoretical description of the RPM required to understand and model possible MFEs through the RPM. An exploration of the magnetic component of RF radiation and its presence in the environment will be conducted (section 3), and selected experimental studies claiming to observe the effects of weak RF-MF in biological systems will be presented and discussed (section 4). Subsequently, the RPM is illustrated in greater detail, and the properties of an SCRP in a biological environment required for an MFE are described (sections 8, 9 and 11). Lastly, an overview of theoretical attempts to study RF-EMF effects using the RPM is provided (section 12). The review presented herein aims to provide an overview of the current knowledge and to guide future explorations while consolidating the understanding of the potential role of the RPM in potential effects of weak RF-EMFs.

## 2 Definitions and Clarifications

Several regimes of frequencies and field strengths must be considered when assessing the effects of magnetic fields on biological systems. This review focuses on low-amplitude oscillating magnetic fields that, due to their different physical properties, are divided into several frequency domains. The potential influence of the electric field component will not be the focus of this manuscript, as the scope is limited to magnetic field interactions relevant to the radical pair mechanism, which is not directly sensitive to electric fields. To guarantee a clear and systematic discussion of the experimental results, this review will use the definitions found in Table 1 regarding MFs, arranged with increasing frequency ν domains.

**Table 1:** Frequency Regimes and Respective Abbreviations Defined for This Review

abbreviation	meaning	frequency range
SMF	static magnetic field	0
ELF-MF	extremely low-frequency magnetic field	0 < ν < 300 Hz
RF-MF	radiofrequency magnetic field	100 kHz < ν < 300 GHz

A definition of SMF and ELF-MF as “weak” is applied for magnetic flux density amplitudes below 100 μT. RF-MFs will be called “weak” when the root-mean-square field strengths are below about 1 A/m (corresponding to flux densities of 1.25 μT in nonmagnetic matter). Furthermore, note that the meaning of the “intensity” is often (inaccurately) used to describe the flux of an MF, in contrast to its square, as it is usually used in the quantum biology community. The term “weak MF” will be used throughout the article for statements about such field strengths, including all of the frequencies in Table 1.

## 3 Radiofrequency Radiation and Anthropogenic Sources

To understand the potential role of the RPM in the context of possible effects of anthropogenic low-amplitude oscillating MFs on biological systems, it is essential to understand the characteristics and origins of such fields. In today’s rapidly advancing technological era, EMFs have become ubiquitous in urban environments, serving as essential tools for communication and, at times, contributing to electronic background noise, emitting a wide range of frequencies in the environment.^1,2,4^

RF-EMFs are among the most notable and intensively discussed forms of anthropogenic radiation.^19,79^ Several properties, such as frequency, field strength, and polarization, must be considered when studying the possible interactions of RF-EMFs with matter. EMFs consist of two field components: the electric field (*E⃗*) and the magnetic field (*H⃗*), each describing distinct aspects of the EMFs interactions with matter.^79,80^ In particular, the magnetic field component directly relates to the magnetic flux density (*B⃗*). In the literature, however, it is common to name the magnetic flux density *B⃗* as the magnetic field in terms of the RPM. Furthermore, in a diamagnetic matrix such as water, the magnetic field strength and flux density are directly related, i.e., differ only in units.^81^

RF-EMFs are defined as electromagnetic waves within the frequency range of 100 kHz to 300 GHz.^1^ They commonly originate from the acceleration of electric charges in oscillatory circuits; however, several anthropogenic sources exist that emit RF-EMFs in various frequency domains. For instance, within the 1–10 MHz range, various electronic devices like television, car ignitions, and AM radios emit waves into the surrounding environment.^2^ Table 2 compiles familiar RF-EMF sources and their respective frequencies. Notably, the intensity and magnitude of these RF-EMFs vary significantly depending on the distance from the radiation source.

**Table 2:** Major Sources of Exposure to RF Radiation^a^

source	uses	frequency domain
dielectric heaters	heat, form, seal, or emboss dielectric materials	1–100 MHz, 27.12 MHz
induction heaters	heat, harden, forge, weld, anneal, or temper conductive materials	250–500 kHz
microwave heaters	dry, cure, heat, or bake materials	915 and 2450 MHz
welding	RF-stabilized welding	0.4–100 MHz
radar	acquisition, tracking, and traffic control	1–15 GHz
communications	fixed systems: satellite communications; microwave; high-frequency, tropospheric scatter	0.8–15 GHz
	mobile systems: two-way transceivers; wireless devices; two-way pagers; Code Division Multiple Access (CDMA)	27 MHz–3 GHz; 2G: 800 MHz; CDMA: 850, 900, 1800, 1900 MHz; 3G: 800–900 or 1700–2100 MHz; 4G: 700, 1700, 2100, 2500–2690 MHz; 5G: <6 GHz
broadcasting	AM radio, FM radio, CB radio, VHF TV, UHF TV	AM radio: 535–1605 kHz; FM radio: 88–108 MHz, CB radio: 27 MHz; VHF TV: 54–72, 76–88, 174–216 MHz; UHF TV: 470–890 MHz
diathermy	shortwave or microwave diathermy in physiotherapy	13, 27, 915, or 2450 MHz
radiofrequency identification	tracking and identification of objects	120–140 kHz, 13.56 MHz, 868–928 MHz

a Data were extracted from refs 82–85 .

In general, the magnitude of RF-EMFs decreases with distance, so even though there are many sources in the environment, proximity to a particular source (e.g., next to a radio broadcast antenna) typically dominates the exposure.^80^ For example, the maximum MF field strength of anthropogenic EM noise at any single frequency (10 kHz to 5 MHz) measured around the University of Oldenburg (Germany) is only 0.1–50 nT.^58,59^ These field strengths are orders of magnitude smaller than the MF strengths that are usually associated with discernible effects in the RPM for typical systems.^54,86,87^ Furthermore, the maximum amplitude of some RF-EMF sources (i.e., mobile radio base stations) can be found up to 200 m from the emission source.^88^ The interaction between RF-EMFs and biological matter depends significantly on various factors such as frequency, amplitude, modulation, and duration of exposure, in addition to properties of the receiving material such as size, shape, and dielectric parameters regarding its composition in the EMF.^82^

It is crucial to note that the penetration depth of RF-EMFs into biological tissues is frequency-dependent, varying from meters in the MHz range to millimeters in the tens of GHz range. This requires a precise measurement of field strengths within the biological medium. Exposure to RF-EMFs is commonly characterized by different quantities such as the specific absorption rate (SAR, in W/kg), the specific absorption (SA in J/kg), the field strength (*E* in V/m, *H* in A/m, *B* in T), or the power density (*S* in W/m^2^).^82^ For a comprehensive treatment of RF dosimetry and safety standards, the reader is directed to previous work.^89–91^ The direct conversion between the characterizing quantities is not always trivial, and precise knowledge of several parameters of an exposed system and the corresponding radiation is required.^82^ The characteristic metrics are divided into body/tissue-internal or -external parameters.

When studying the effects of RF-EMF on biological systems, it is essential to divide the spatial volume around the source of EMF into the near-field and far-field regions.^4^ The far-field region is located at distances greater than one wavelength of the respective EMF from the source, i.e., an RF-EMF of 1–10 MHz frequency at distances approximately 30–300 m.^4^ In the far-field region, the magnetic and electric field components are in phase and propagate orthogonally. Measurement of one field is sufficient to determine the field strength of the other.^80^ Under far-field conditions, the electric and magnetic field strengths decrease with increasing distance *r* usually following 1/*r* from the source.^80^

The situation is more complex in the near-field region as the magnetic and electric fields have to be considered decoupled and are not straightforwardly interconverted due to interference processes near the source. It is essential to measure both field components independently to obtain reliable magnitudes, as a measurement of the general power flux (usually in W) alone cannot adequately describe the conditions within the near-field region.^4^ Note that most biological experiments related to RF are conducted within the near-field region.^4^ The commonly used quantity as a metric in these experiments is the power flux.

Furthermore, in the near-field region, two domains must be considered when measuring the field strength: *reactive* and *radiative* near-fields.^80^ The *reactive* near-field region contains stored nonradiating energy (quasi-static fields) and is closest to the source of EMF fields, where the magnetic and electric fields are considered decoupled (see Figure 1).^80^ The amplitudes and phases of the electric and magnetic fields vary significantly with distance *r* from the source in the reactive near-field region (e.g., the amplitudes decrease with 1/*r* ^3^). The ratio of amplitudes and phases of the fields *E⃗* and *B⃗* is not constant and cannot be easily estimated without detailed knowledge of the structure and shape of the EM source.^80^ It is crucial to independently measure the field strengths of *E⃗* and *H⃗* or *B⃗* at each point of interest.

**Figure 1:**
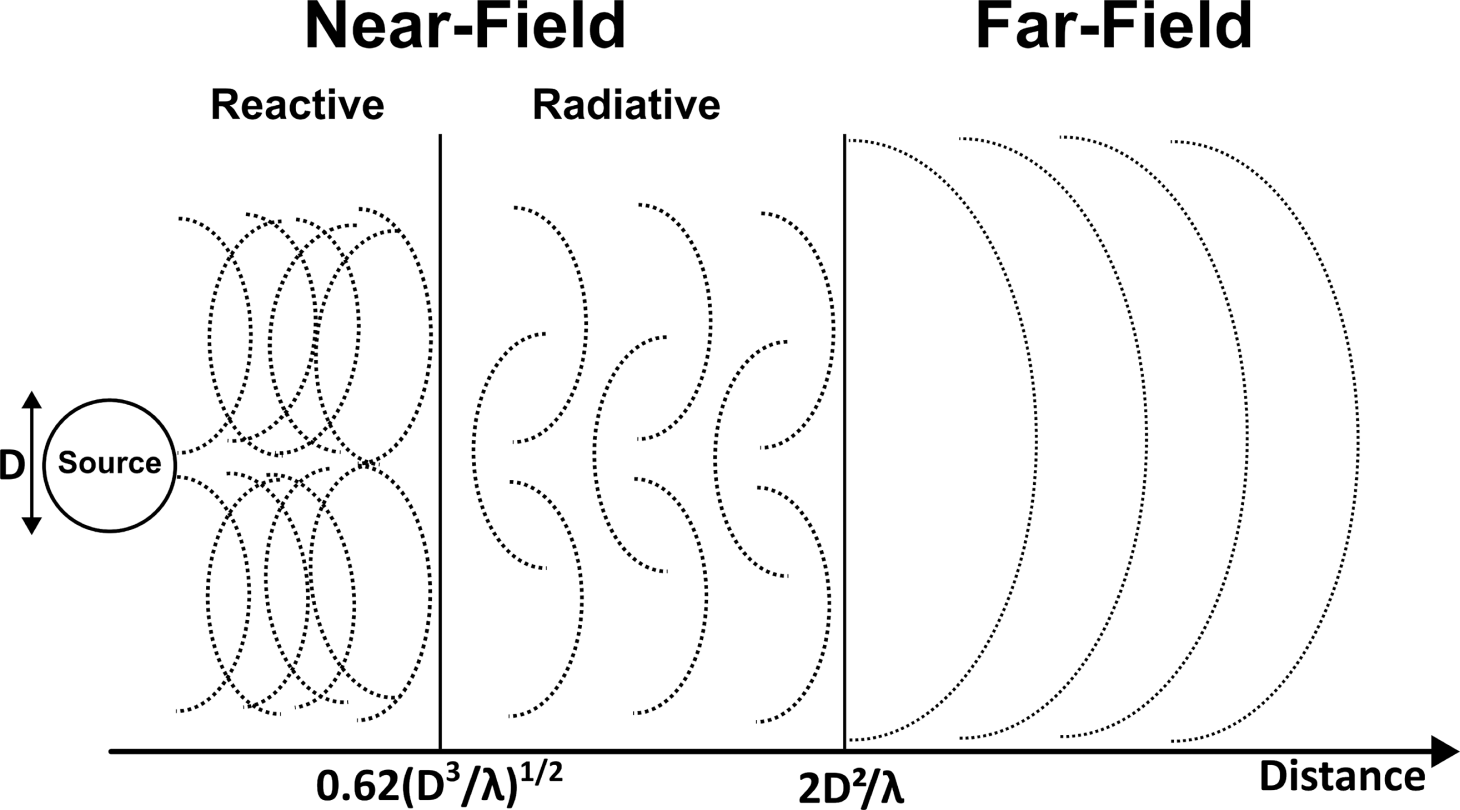
Illustration of different field regimes concerning the source distance, showing approximate EM wavefront structures (dotted lines) due to interference of electric and magnetic field components. The near-field region includes both reactive and radiative contributions, where electric and magnetic fields are not necessarily in phase and can be spatially decoupled. The complex wavefronts in the near-field arise from this interference (individual components of the electric or magnetic fields in the wavefront are not displayed here). The regions can be approximated through the length *D* of the source (treated as an antenna) and the wavelength of the radiation λ.^92^ The amplitudes of the field components fall off with different behaviors depending on the distance *r*.^80^ For the far-field, the amplitude commonly decreases with 1/*r*; for the radiative near-field, the amplitude falls off as 1/*r* ^2^; and for the reactive near-field region, the amplitude decreases with 1/*r* ^3^.^80^ In the near-field regions, the magnetic and electric field components can be considered as decoupled due to interference processes near the radiation source, as illustrated by the dotted lines.

The second type of near-field region, the *radiative* near-field region, is farther away (depending on the wavelength) from the source.^80^ In this region, the spatial distributions of *E⃗* and *H⃗*/*B⃗* are better predicted because the radiation does not contain reactive field components from the source antenna, but the far-field radiation pattern of the source is not yet fully formed. Generally, the field strength does not diminish in direct proportion to increasing distance from the source but more rapidly in the two near-field domains. Thus, measurements of near-field EMF properties should be made at frequent spatial intervals to study possible correlations between biological effects and EMFs, as significant amplitude variations may occur over small spatial regions.^80^ Preliminary field parameter measurements are vital to estimate the spatial gradients in the region of interest. Depending on the spatial extent of the biological material studied, significant field strength variations can be found within the sample and must be considered when evaluating possible biological effects. The precise measurement of such field properties in the context of biological systems remains an active topic of research.^93^

In anthropogenic RF-MFs and their potential effects on biological systems, among others, two different measurement procedures, the frequency selective and the *broadband*, must be distinguished. The frequency selective procedure limits the EM wave to a small range of frequencies, allowing a direct field magnitude measurement of the nearly sinusoidal amplitude.^4^ The *broadband* procedure spans a larger spectral domain where only the total power density can be measured. Accurately evaluating and adjusting the properties of RF-MFs within an experimental setup studying RF effects on biological systems can pose a challenge, particularly when dealing with exceptionally weak EMFs.^22^

It becomes apparent that the precise knowledge of the involved EM source and the corresponding field properties are equally challenging to evaluate, as is the quantification of possible biological effects with a biological system exposed to weak RF-EMF. Without precise knowledge of the field parameters within the spatial volume in which a biological effect is being investigated, it is impossible to reliably assess the potential impact of RF-EMF in a proposed study. Therefore, any biological research on magnetic field effects should include a comprehensive dosimetry assessment for a systematic and reproducible study.

## 4 Suggested Biological Effects Linked to RF-MFs

The influence of RF-EMFs on biological systems is only a subpart of an increasingly prominent, yet controversial, subject of weak EMF effects in biological processes. The plausibility of effects of weak RF-EMF is assessed and discussed continuously in the scientific community.^94–97^ In this review, only selected examples of the underlying research are presented due to its vast scope and the detailed and systematic listing by other reviews.^10,11,94–97^ Rather than providing an exhaustive overview, the selected biological systems serve to orient the reader toward the key experimental approaches taken and to construct a coherent picture of the systems and mechanisms that may be involved. It should also be noted that we do not claim domain expertise in all the experimental studies cited, particularly in the areas of animal behavior and other complex biological assays. Our summaries and discussions of such studies should therefore be understood within this context. Where appropriate, we emphasize theoretical considerations that might guide or constrain the interpretation of these experimental findings.

Figure 2 illustrates the increasing number of publications on RF radiation and health (A), biological effects (B), radicals (C), and reactive oxygen species (D). The growing interest in RF-induced biological effects is fueled not only by the frequently discussed concept of magnetoreception in migratory species^56–59,98^ but also by a dense and partly contradictory landscape of *in vitro* studies in various biological systems. Among several theoretical attempts,^66,99,100^ the RPM stands out as a leading candidate to explain the observed effects of external MFs on biological systems.

**Figure 2:**
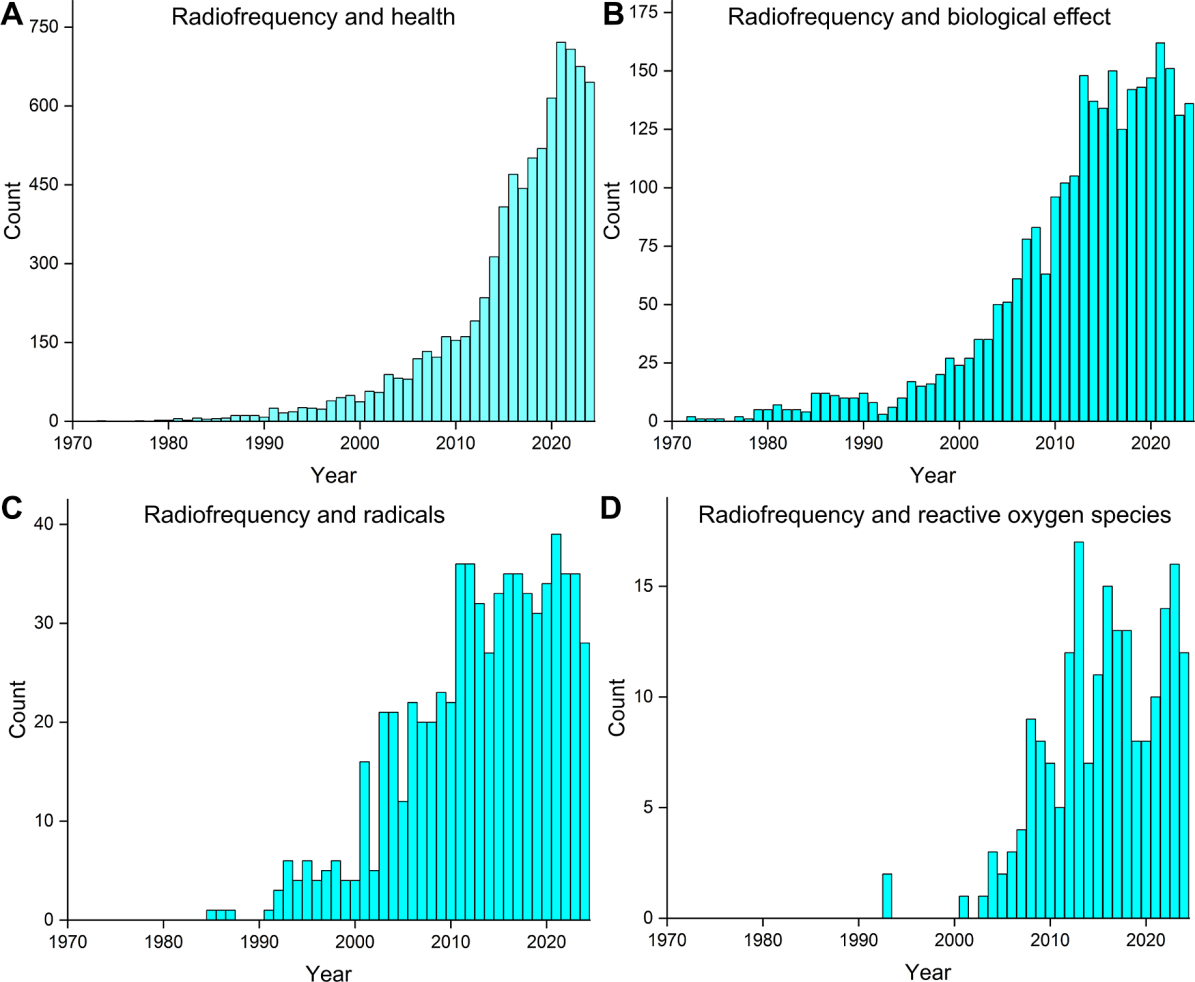
Number of publications found at the NCBI PubMed database^101^ for combination of different keywords. There is a steady increase of published articles considering radiofrequency in terms of (A) health, (B) biological effects, (C) radicals, and (D) reactive oxygen species. Data were obtained on November 11, 2024.

Zadeh-Haghighi et al.^10^ and Wang et al.^11^ recently conducted a noncritical review of studies claiming biological effects that might be correlated to the RPM as a potential factor in their experimental outcomes. The authors surveyed a range of biological systems, including cryptochromes, stem cells, neurons, brain tissue, and DNA, examining their responses to SMFs (≤250 mT), hypomagnetic fields (approximately 0 μT), and extremely low-frequency MFs in different RMS magnetic flux densities.^10,11^ A notable result of these studies is the observed variation in ROS production under the influence of weak MFs.^9,70–72^ Elevated ROS levels, while sometimes beneficial for an organism,^102^ can primarily induce oxidative stress, resulting from an imbalance between ROS production and the system’s ability to neutralize these reactive molecules and repair associated cellular damage.^9^ The possible increased formation of ROS, in addition to reactive nitrogen species, under the influence of weak EMFs has been extensively reported, notably by Georgiou^66^ and Wang et al.^11^ These studies indicate that the presence of transition metals, liberated from enzymes during oxidative processes, catalyzes the formation of ROS through the Haber–Weiss/Fenton reaction.^66,103^ For example, the [Fe–S] cluster in mitochondrial aconitase has been considered to be a potential source of the required transition metal ions.^104^ Cells exhibiting abnormal proliferation and elevated free iron concentrations might have been subject to oxidative stress as a result of the Haber–Weiss/Fenton reaction, whereby effects are particularly severe under ROS-associated redox control.^9^

Further experimental findings indicate that oxidative stress, attributed to the influence of EMF on the Haber–Weiss/Fenton reaction (without proposing a precise mechanism), occurs across the EM spectrum.^10,66^ For example, a study documented the effects on Fe^2+^-treated rat lymphocytes exposed to continuous RF-EMF (930 MHz)^70^ and discussed the involvement of the Haber–Weiss/Fenton reaction.

Additionally, various biological systems pertinent to human physiology have been claimed to be adversely affected by oxidative stress. Notable biological processes, specifically involving RF-EMFs include lipid peroxidation,^78,105–107^ DNA degradation,^108,109^ and influence on proteins and enzymes.^110,111^ However, in most studies of oxidative stress through RF-EMFs, the MF RMS amplitudes were not measured, and only the power density of the RF-EMF source or its SAR was mentioned. For example, two studies reported DNA damage in rat brain cells exposed to 2450 MHz (RF-EMF) with SAR of 1.2 W/kg for 2 h^108,109^ without further dosimetric assessment. These observations were attributed to oxidative stress mechanisms, particularly the formation of ROS through the Haber–Weiss/Fenton reaction.^66^ RF-EMF (400 and 900 MHz with 10, 23, 41, and 120 V m^–1^ field strengths) exposure-induced oxidative stress was also claimed to be observed in plant tissues (duckweed).^112^

Another study observed that 4 h of exposure to 900 MHz RF-EMF from a typical mobile phone led to an 11% increase in blood plasma lipid peroxidation in human volunteers. Specifically, effects on adult males aged between 20 and 25 years were studied;^78^ no RF-EMF field strengths or intensities were reported in this study, allowing no proper dosimetric assessment. Similar increases in lipid peroxidation were also reported in human blood platelets subjected to up to 7 min of exposure to 900 MHz RF-EMF (0.2 W, source in 4 cm distance) from cell phones.^105^ Numerous studies have investigated the effects of RF-EMFs from mobile phones and WiFi devices using rats as test subjects.^106,107,113^ These studies commonly reported increased lipid peroxidation in various tissues of rats, especially when exposed to RF-EMF of 900 or 2450 MHz. However, the lack of dosimetric details again complicates the interpretation of the results. While no theoretical studies on lipid peroxidation, including RF-EMFs, were performed, the possible involvement of the RPM in lipid peroxidation processes and the influence of SMFs were discussed theoretically by Sampson et al.^114,115^

An explanation for the observed oxidative stress through increased ROS formation and the possible action of the RPM (not only due to RF-MFs, but weak MFs in general) has been raised in several studies through the formation of superoxide species. Superoxide O_2_^•–^ is a prominent ROS, often discussed with regard to MF effects through the RPM in biological systems.^10,69,116–118^ The increased formation of O_2_^•–^ and other types of ROS such as H_2_O_2_ was found to occur within transmembrane NADPH oxidases^119,120^ and the mitochondrial electron transport chain (ETC).^121,122^ In the latter case, Sheu et al.^123^ reported that SMFs (0–1.93 mT) can modulate mitochondrial ETC activity, thus enhancing mitochondrial respiration. The authors propose that these effects on mitochondrial activity could be explained by the RPM. Another study claims to observe elevated H_2_O_2_ concentrations in pulmonary arterial smooth muscle cells under the influence of an SMF (45 μT) and an RF-MF (10 μT_RMS_), and directly connect their results to a radical pair between semiquinone flavin and O_2_^•–^.^124^ In a more recent study, Chow et al. investigated the effect of 72 h of RF-EMF exposure (10 μT RMS amplitude at 6.78 MHz) on human umbilical vein endothelial cells.^68^ Here, they demonstrated that exposure to RF-EMF inhibits cell apoptosis and causes an increase in cell number while directly measuring the concentrations of O_2_^•–^ and H_2_O_2_. Furthermore, Chow et al. hypothesized several potential reaction pathways involving the protein enzyme NADPH oxidase that are influenced by the RF-EMF exposure.^68^

However, the role of O_2_^•–^ as a contributor in the RPM has been questioned, particularly due to its rapid motion, which leads to a rapid decoherence of the SCRP due to the spin–orbit coupling of superoxide (see section 11.2.4) and consequently suppresses MFEs.^125^ This skepticism is further supported by the absence of experimental findings showing superoxides at room temperature, especially in areas of high viscosity such as mitochondrial membrane bilayers, as confirmed by EPR spectroscopy.^125^ Remarkably, recent molecular dynamics (MD) studies have indicated that the binding time of superoxides to the electron-transferring flavin protein (ETF) could be extraordinarily long, up to 40 ns.^116^ Similarly, the appearance of bound superoxides was recently studied in MD simulations of the pigeon (*Columba livia*) cryptochrome protein by Deviers et al.^126^ forming a possible radical pair with the protein-embedded flavin cofactor. The simulation findings of the two studies suggest the possibility that the fast rotational effects of superoxides, which diminish magnetic field effects, might be sufficiently suppressed under specific conditions (however, the superoxide has to rotate at least 1000 times less rapidly than it does in aqueous solution,^125^ as found in^126^).

More concrete investigations of the RPM and possible MFEs through exposure to weak RF-MF are found at the experimental level within the topic of magnetoreception.^56–59,98,127–129^ Migratory songbirds rely on the geomagnetic field, approximately 50 μT, for precise navigation to specific destinations.^54,55,130^ The RPM is proposed as a viable mechanism that can explain various experimentally observed features of magnetoreception.^55^ Specifically, the photoreceptor protein cryptochrome was suggested^131,132^ to be the biological origin of RPM-based magnetoreception. Radical pairs are generated through the photoexcitation of the embedded cofactor flavin adenine dinucleotide (FAD), followed by an electron transfer chain with adjacent tryptophan residues.^54,55^ Both experimental and theoretical studies have validated that MFEs (SMF) can be observed in cryptochrome proteins, providing substantiated evidence that the RPM can be a potential mechanism for magnetoreception.^14,54,55,75,76,133,134^ Furthermore, the work on cryptochrome has spawned several studies of possible MFEs on ROS generated by the flavin photochemistry.^126,135–138^ The possible effect of weak RF-MFs on the navigational sense of migratory songbirds was investigated by several groups,^56–59^ indicating that exposure to specific frequency bands of weak RF-MF has a disruptive effect on the orientation of songbirds. For example, a recent study by Leberecht et al. examined the impact of weak broadband RF-MFs (∼2–4 pT/Hz^1/2^), no SAR was conducted) on blackcaps.^56^ The study identified a disruption threshold of around 116 MHz. Frequencies above the threshold do not disorient migratory songbirds.^56^ The work of Leberecht et al., along with previous research,^57–59^ defines a relevant frequency domain of weak RF-MF that extends from 100 kHz to 116 MHz, consistent with the eigenvalue spectrum of the spin Hamiltonian of a flavin-containing radical pair. In addition, disoriented behavior was found for other species. The magnetic orientation of an amphipod (*Gondogeneia antarctica*) was suggested to be influenced by a 10 MHz MF with amplitudes as low as 20 nT,^61^ furthermore discussing that the effect may extend to higher frequencies.

It becomes apparent that there is a manifold of experimental results that claim an observable effect of RF-(E)­MFs on biological systems and connect it with the RPM. However, several of these studies were never replicated and did not provide enough information on the experimental setup and dosimetric evaluation. Due to these and other systematic problems, critiques of the experimental setups and the reproducibility of many experiments arose, which will be elaborated on in the next section.

## 5 Criticism of Experimental Studies

Although there are many experimental studies that claim to demonstrate the effects of weak RF-MFs on several relevant biological systems, the topic remains controversial.^139^ A significant reason is the often lacking reproducibility of many studies, leading to a questionable reliability of published works.^22,139–141^

Berg^22^ stated that there are only a few cases of reproducible EMF windows for the three parameters: (a) frequency, (b) amplitude, and (c) exposure duration. In addition, other environmental factors such as temperature, conductivity, osmolarity, nutrition medium, special additives, etc., play a crucial role in producing reliable data and are often missing from representative studies.^22^ The environmental conditions under which the highly sensitive experiments are executed lead to different results in several cases.^22,142–144^ For example, the claim that the cryptochrome-mediated behavior of *Drosophila* through weak MFs (SMF; 0–300 μT)^142,143^ was recently called into doubt by an extensive study by Bassetto et al. studying 97,658 flies.^144^ The authors furthermore emphasized the importance of publishing negative results. While Bassetto et al.’s findings raise questions about *Drosophila*’s magnetosensitivity in specific behavioral paradigms, their conclusions have been contested by Kyriacou^145^ and Reppert,^146^ and a substantial body of research continues to support the existence of a magnetic sense in *Drosophila* (see references within^142–146^ ). The responses by Kyriacou and Reppert were furthermore addressed by a follow-up response by Bassetto et al.^147^ This studies and replies raise important points about the correct use of statistics, the required number of replicas, and overall demonstrate the difficulty of reproducible experimentation when involving complex subjects, such as animal behavior. In addition to the nonexistent reproducibility and the lack of presentation of experimental parameters, several studies are inconsistent, e.g., in the case of studies for RF-induced cancer promotion.^82^

Binhi and Rubin discussed in detail why the reproduction of nonspecific effects (discussed by authors as biological effects where the magnetic receptors are unknown) is challenging.^140^ The authors mention that in 21 incubators studied for biological research, the inhomogeneity of MF reached hundreds of microtesla per 10 cm.^140^ Furthermore, nonspecific effects in magnetobiology depend on factors which are difficult to control. In addition to the MF and the electric field properties, these include temperature, humidity, pressure, illumination, the rate of their changes, and chemical, physiological and genotypic factors.^140^ Even small changes in one of these factors can produce an observable response, and thus a high amount of randomness is inherently present. As an example, the authors emphasized that the responses of organisms to geomagnetic disturbances (where the amplitude of change is 1/100 of the quasi-stationary geomagnetic field) are reported; however, researchers in biological laboratories usually do not monitor the level of geomagnetic variations.^140^

For SMF strengths smaller than the geomagnetic field (∼50 μT), Adair demonstrated that the chemistry of an SCRP could only be affected under special conditions,^148^ making the overall finding of an RPM-based biological effect due to MFs with field strengths much smaller than the geomagnetic field unlikely. Hore extended Adair’s discussion and claimed that it is unlikely that extremely low-frequency MFs of 50 or 60 Hz with an amplitude of <1 μT will affect radical pairs significantly.^47^ Hore emphasized again that only a few of the experimental observations have been replicated independently.^47^

Gauger et al. stated that in the case of magnetoreception studies, interactions of an SCRP with weak oscillating magnetic fields must involve an unusually long SCRP coherence lifetime over several microseconds and a remarkable resistance of the SCRP against external noise introducing spin relaxation through decoherence.^73^ It was emphasized that in 2006, for a N@C_60_ fullerene (which is expected to be a much more rigid system than proteins such as cryptochrome), an electron decoherence time of 80 μs^149^ was found, making a coherence lifetime of >10 μs for an SCRP in a biological noisy environment unlikely. Nevertheless, in more rigid systems such as nitrogen-vacancy (NV) diamond centers much longer relaxation times of up to milliseconds^150^ and the structural integrity of biological systems is often unknown.

For SCRPs including the frequently mentioned superoxide,^10,69,116–118^ it has been demonstrated that an MFE is unlikely due to the rapid tumbling of the linear molecule (O_2_^•–^) and the inherent spin–orbit coupling inducing fast spin relaxation.^125,151^

Thus, although several experimental studies claim to reveal (weak) biological effects induced by exposure to RF-MFs, the underlying mechanisms of these effects remain unclear. In recent years only a few theoretical studies of the effects of very weak (<10 μT) RF-MFs on the SCRP were conducted.^73,75^ All concluded that either the lifetime of the SCRP must be extraordinarily long or the field strength of the RF-MF must be higher than expected, creating a gap between theory and experimental observations.

To advance the credibility and reproducibility of experimental studies in this area, it is crucial to obey a minimal set of methodological standards. First, confounding factors must be minimized through randomized, double-blind study designs and rigorously enforced control conditions. This includes conducting the field-free baseline experiments under otherwise identical environmental conditions, tightly regulating parameters such as temperature, light exposure, humidity, and chemical composition of the sample environment.^140^ Second, magnetic field exposure must be fully characterized, including static and time-varying components, field homogeneity, and shielding from environmental electromagnetic noise, and controlled. The choice of field intensities and frequencies should be directly linked to mechanistic predictions, especially those from the RPM, which suggests that observable effects are only expected within specific frequency regions, and when radical pair lifetimes and decoherence time scales are sufficiently long.^47,73^ The number of replicas must suffice to draw meaningful conclusions in view of the expected effect sizes. Without adherence to these minimum criteria, the risk of irreproducibility, misinterpretation, or overstatement of effects remains high. Defining and adopting such standards would represent a major step forward in the maturation of magnetobiology as a discipline.

Before exploring and discussing these theoretical attempts, it is crucial to understand the theoretical background and foundation of the RPM, elucidate the properties of an MFE, and outline the theoretical framework based on quantum dynamics, providing a rigorous overview of the viability of sizable effects. Therefore, the subsequent sections delve into each of these aspects to equip the reader with comprehensive knowledge before discussing the limited theoretical studies undertaken in recent years to comprehend the experimental observation of a biological effect induced by weak RF MFs.

## 6 Why Is the RPM an Attractive Hypothesis?

Although other theories, such as the cyclotron resonance or the involvement of magnetic nanostructures, i.e., magnetite, exist^99,152^ to explain magnetic field effects in biological systems, RPM has gained significant attention as a potential explanation. However, the question arises as to why the RPM has gained such popularity over the past few decades. The first reason is that the underlying effect induced via weak MFs appears likely to originate from within the molecular scale of a biological system. Furthermore, larger structures such as whole proteins or lipids as building blocks of a biological system such as cells (see Figure 3) are unlikely to be directly structurally affected when MFs with flux densities <100 μT are present. Unpaired electrons, as the smallest building block in a biological process, can, in principle, be affected by such weak MFs (see Figure 3), raising the interest of RPM as a potential explanation for the claimed biological effects. The interaction of radicals with MFs is described through the quantum-mechanical nature of electron spin, which induces a magnetic moment capable of interacting with MFs. The RPM is built on this very feature of nature and thus provides a reasonable foundation as an explanatory tool for magnetic field effects in biological systems.

**Figure 3:**
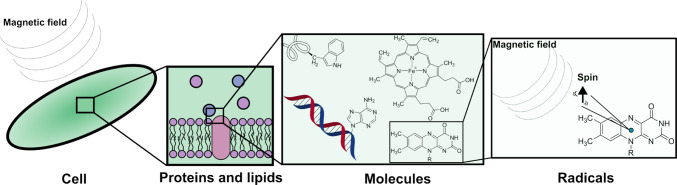
Illustration of the emergence of magnetic field effects in biological systems through the RPM. Within a biological system (e.g., a cell), many functional protein and lipid structures exist that include molecules able to form radicals through electron transfers or chemical reactions. Radicals have unpaired electrons, which can, in principle, interact with weak MFs by precessing around the external MF. However, due to the magnetic interactions with other spins and the constant motion of the atoms within the molecules (an inevitable effect in proteins), the spin dynamics is modulated, and the system evolves toward thermal equilibrium, damping the effect of weak external magnetic fields.

First independently proposed by Closs^153^ and Kaptein and Oosterhoff,^45^ the RPM has further compelling attributes supporting its relevance and application in biological systems. The RPM plays a central role in spin chemistry and has been experimentally verified in several studies.^154–160^ For example, the SCRPs of organic molecules, including dyad systems,^161–163^ have been extensively studied, verifying the RPM as the working principle at moderate MF flux densities (>0.1 mT). Radical pairs are, furthermore, found in several biological systems. Notable examples include the special pair of the photosynthetic system in various organisms,^164–166^ the mutated LOV domain protein,^157,158,160,167^ the photolyases for DNA repair,^168^ coenzyme B12-dependent enzymes,^169–171^ and the cryptochrome proteins in avian species and other animals.^43,54,172^ Electron transfer cascades, which often involve radical pairs, are integral to multiple human-relevant systems, such as NADPH oxidase^119,120^ and respiratory complexes within mitochondria.^116,173^ These cascades are critical for various biochemical processes, including energy production and cellular defense mechanisms. The involvement of radical pairs in these essential processes underscores the broader significance of the RPM in biology.

Radical pairs in biological systems have been detected by magnetic resonance methods such as chemically induced dynamic nuclear polarization (CIDNP)^87,158–160^ and electron paramagnetic resonance (EPR).^164,165^ Furthermore, the CIDNP experiments are only explicable due to the existence of the RPM.^45^

It becomes evident that the RPM is an attractive hypothesis because of its experimental verification, presence in biological and human-relevant systems, detectability through advanced techniques, and ability to explain sensitivity to weak MFs. Together, these factors make the RPM a compelling framework for understanding the role of magnetic fields in biological systems.

However, it remains unclear how MFs of low intensity (in the nanotesla regime) can affect the RPM in the context of a fluctuating environment within a protein or a cell that perturbs spin interactions within radicals and produces fast spin relaxation. While artificially synthesized dyad systems, where the RPM was studied several times, allow for rigorous frameworks in which the influence of a weak MF may be felt by a radical pair within a living system, the usual fluctuations through thermal motion challenge the RPM hypothesis heavily when concerning weak/very weak magnetic fields. Current research focuses on the possibility that the RPM could be the working mechanism for the effects of weak MFs in biological systems and, if so, how the RPM can function within a fluctuating environment. Within this research, topics such as specially designed protein structures, which are more rigid, are discussed.^77^ To get a deeper insight into the apparent problems in a biological environment, however, it is crucial to understand the physics of the RPM itself. Thus, the following sections will delve into the details of the RPM, emphasizing its potential and flaws and providing a rigorous theoretical background to ensure a comprehensive understanding of the current state.

## 7 Alternative Theories to the RPM

Although RPM has emerged as the leading hypothesis for explaining weak MFEs in biological systems, several alternative theories have been proposed. These alternative theories attempt to account for magnetobiological effects through different mechanisms. However, these alternative theories face substantial limitations, raising questions about their validity as comprehensive explanations for the observed phenomena. Nevertheless, the brief introduction of potential alternative theoretical frameworks is conducive to comprehending the complexity of interactions between weak oscillating fields and soft matter.

One alternative suggestion is the cyclotron resonance.^99,174^ Cyclotron resonance occurs when a charged particle (such as an ion) moves in a magnetic field and resonates with an applied oscillating electric field at a frequency equal to its cyclotron frequency, $f_{c}=\frac{q|\mathrm{B⃗}|}{2\pi m}$ , where *q* is the charge of the particle, |*B⃗*| is the magnetic field strength, and *m* is the particle mass. In principle, this resonance could affect cellular processes by disrupting ion transport or influencing membrane potentials. Thus, cyclotron resonance is hypothesized to interact with calcium, potassium, or other biologically relevant ions, potentially influencing cell signaling.

However, cyclotron resonance theory encounters several challenges that limit its applicability to biological systems. For resonance to occur, the magnetic field and ion velocities must align significantly, which is difficult to achieve and maintain in living organisms. The cyclotron resonance theory assumes that ions within cells follow predictable, coherent paths that resonate with external fields. However, biological conditions, such as the thermal motion of ions, the cellular environments, and the impact of complex biological structures, significantly disrupt such coherence.^175,176^ Furthermore, the applicability of the cyclotron resonance theory to weak fields remains uncertain, as the field strengths required to achieve cyclotron resonance in biological environments are typically much higher than those found in most natural or anthropogenic magnetic environments.^175–177^ Consequently, while cyclotron resonance may offer a theoretical basis for magnetic effects on free ions, the stringent requirements for resonance make it unlikely to be a reliable explanation for weak magnetic field interactions in complex biological systems.

Another alternative theory proposes that magnetic nanostructures, specifically magnetite (Fe_3_O_4_), could be biological sensors for external magnetic fields. Magnetite particles have been discovered in various organisms, including migratory birds and some mammalian tissues, where they are hypothesized to contribute to magnetic field sensitivity.^152^ A prominent organism is the magnetotactic bacterium, which is an entire cellular structure evolved for magnetic sensing by possessing magnetic particles in form of a compass needle within its body.^178,179^ These particles exhibit large magnetic moments that, in principle, could influence nearby biological processes by aligning or reorienting them in response to an external magnetic field.^14^ This theory, for example, suggests that the rotation or translation of magnetite particles within cells could provide directional cues for organisms that rely on magnetoreception, such as migratory birds.^152^

Despite its intriguing foundation, the magnetite-based theory also faces considerable limitations in explaining weak magnetic field effects, especially in humans and nonmigratory species where magnetite is present only in trace amounts or may not be functionally significant. To serve as reliable biological sensors, magnetite particles must be strategically organized, structurally integrated within cells, and protected from disruptive biological noise.^14^ These conditions are rarely observed outside of the specialized sensory organs in magnetoreceptive animals. In many cases, magnetite particles are sparse, disorganized, or sequestered in locations that would prevent their interaction with cellular signaling pathways.^180^ Additionally, most observed biological responses to magnetic fields (e.g., RF exposure) occur at field strengths far below the threshold at which magnetite particles in the size of nanometers would exhibit notable physical reactions. Furthermore, it is questionable whether the frequency domains of radiofrequency radiation align with the frequencies required to interact with the magnetic moments of iron-based nanoparticles. As a result, although the magnetite hypothesis offers a plausible mechanism for magnetoreception in some animals (such as bacteria or unicell algae),^180^ its utility in explaining MFEs in a broad range of biological systems, including humans, remains doubtful.

Although cyclotron resonance and magnetite-based sensing present alternative pathways for understanding MFEs, each theory has limitations that restrict its applicability in explaining weak magnetic field interactions, especially for weak radiofrequency radiation, in a broad range of biological systems. These alternative theories often require precise conditions or assume idealized cellular environments, which are rarely achievable in living organisms. As a result, these limitations, furthermore, reinforce the appeal of the RPM as a leading hypothesis. Nevertheless, further research is essential to rigorously test each of these theories and determine whether a unified model of magnetobiology can be developed.

## 8 The Radical Pair Mechanism for Weak Fields

Understanding the RPM and its complexity is mandatory to understand the potential effects of weak RF-MF exposure on the dynamics of an SCRP and, thus, the change in the chemical reaction yield of possible products. Its functionality fundamentally relies on the quantum-mechanical nature of electron spin, which in terms of mathematical structure is analog to the quantum-mechanical angular momentum. The electron spin *S* is quantized (*S* = 1/2) and can be described as a linear combination of two states labeled by the spin magnetic quantum number *m*_*S*_ with value 1/2 or −1/2. The core of the RPM lies in forming a spin-correlated radical pair, which leads to a new set of quantum-mechanical states through the entanglement of two electron spins. Such an SCRP may be formed through different processes: *geminates* are formed through light-induced excitation followed by electron transfer, hydrogen transfer, or homolytic cleavage of chemical bonds, and *F-pairs* are formed through a random encounter of two radicals and a spin-selective recombination reaction, leaving the spin states that cannot recombine in a correlated state (see Figure 4).^44^ Upon the formation of an SCRP, four distinct quantum states for the electron spins are possible. The first is the singlet state, denoted by |S⟩, with a total spin quantum number of *S* = 0 and a multiplicity of 1. The other three states—|T_+_⟩, |T_0_⟩, and |T_–_⟩—collectively form what is known as the triplet state, each with a total spin angular momentum of *S* = 1.^44,54^ These four different spin states are accessible for the SCRP, and steady interconversion between these states is possible as long as coherences between the states are present.^46^ In the RPM, spin interactions (condensed in the spin Hamiltonian *Ĥ*, Figure 4) are crucial in determining the energy differences and the coherent interconversion between the four possible states. For example, the exchange interaction, represented by the coupling constant *J*, and the Zeeman interaction with an external MF of magnitude |*B⃗*| may significantly alter the energy difference between states (see Figure 5). When the energy difference between the spin states is sufficiently small, the interconversion between SCRP spin states becomes possible. This phenomenon is, for instance, pronounced during instances of energy level crossing (see Figure 5, |S⟩ and |T_–_⟩). In the absence of an external MF, the coherent interconversion between the S and T states of the SCRP is determined only by its intrinsic spin interactions, most prominently the hyperfine interactions with surrounding nuclear spins.^46^

**Figure 4:**
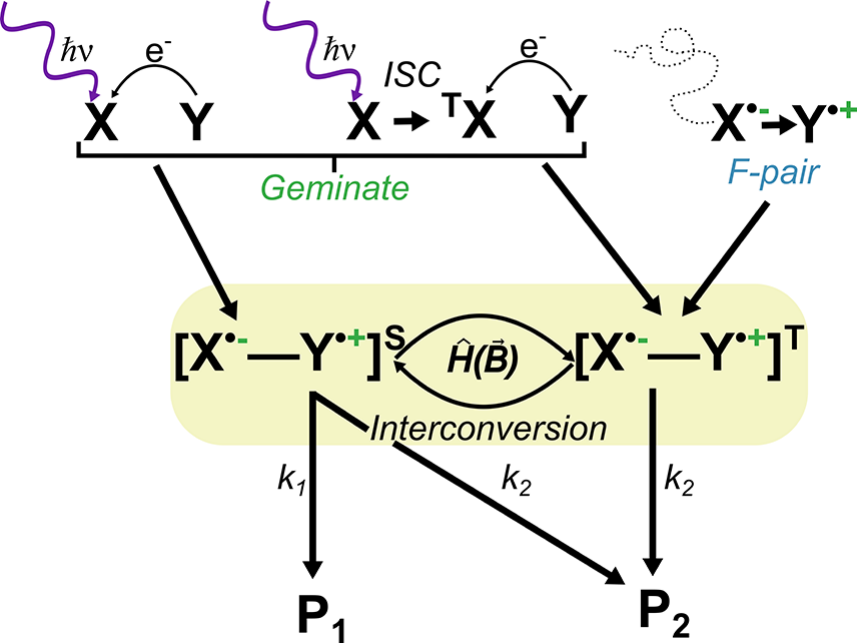
Illustration of the radical pair mechanism. Here, the spin-correlated radical pair (SCRP) is formed through a light-induced electron transfer of diamagnetic molecules, Y and X, leading to a *geminate* SCRP. The SCRP can be singlet-born (singlet = S) or triplet-born (triplet = T) depending on the properties of the photoexcited molecule X. If X’s intersystem crossing (ISC) process is faster than the electron transfer, a triplet-born SCRP will be generated. Note that geminates can be formed through other not photoinduced processes such as electron transfers, H-transfer or bond homolysis. Alternatively, random encounters and recombination of two radicals lead to *F-pair* SCRP formation. The SCRP may undergo interconversion between the S and T states, directed by the system’s Hamiltonian (*Ĥ*), including all interactions, such as the Zeeman interaction with an external MF (*B⃗*). Spin-selective reactions may emerge in a system, i.e., the recombination of the SCRP is often only allowed in the S state as illustrated by the reaction rate constant *k*_1_ (charge-recombinaton was, however, also found in form of triplet–triplet annihilation^181^ for SCRPs). In contrast, other reactions, e.g., forward reactions with other molecules, are spin-independent (reaction rate constant *k*_2_). An external MF alters the ratio between the S and T states, leading to a change in the yields of the products P_1_ and P_2_.

**Figure 5:**
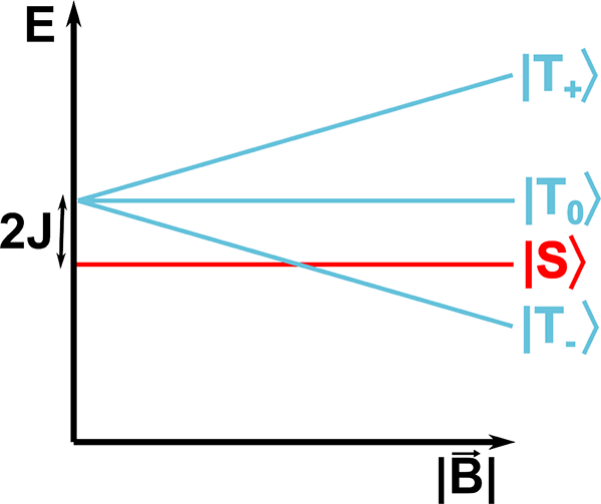
Change of the SCRP energy levels when exchange interaction *J* and an external MF |*B⃗*| are considered. The exchange interaction changes energy differences between the singlet state |S⟩ and the triplet states |T_+_⟩, |T_0_⟩, and |T_–_⟩. The external MF splits the three triplet states.

Another critical aspect is the chemical nature of spin-dependent reactions (see Figure 4). Commonly, the recombination of a geminate singlet-born SCRP is a spin-selective reaction that can occur only in the singlet state. Alternatively, there may be electron transfer processes that will preserve the total angular momentum of the system, which could lead to a spin-selectivity for reactions only occurring in a triplet state. Additional reactions, such as diffusion or interactions with a third, diamagnetic reactant, which are not spin-selective, may occur. As a result, both spin-selective and spin-independent reaction channels are possible, as illustrated in Figure 4 with the two reaction rate constants *k*_1_ and *k*_2_, respectively. Interacting with an external MF alters the interconversion between the singlet state |S⟩, and the triplet states |T⟩ and, thus, the product yields between spin-selective and spin-independent products. An important property for the observation of an MFE through the interaction with an external MF is that an asymmetry between the interactions of the electron spins occurs. For example, the two electron spins have different *g* values (Δ*g*) interacting differently with an MF leading to an emergence of an MFE at high MF amplitudes (∼100 mT).^182^ Furthermore, the coupling of the electron spins to different nuclear spins via hyperfine interactions will also emerge in an MFE when an external MF is applied due to the efficiency of the S–T mixing being reduced by the MF.^46^ These MFEs are known as the “normal” MFE.^46^

In studies of SCRPs within biological systems influenced by anthropogenic RF-MFs, weak MFs (much weaker than, e.g., the hyperfine interactions with nearby nuclear spins) are the focus. The appearance of an MFE at weak MFs is known as the low-field effect (LFE), whose understanding requires more context, which will be provided in the next section.

## 9 The Low-Field Effect

The low-field effect emerging through the RPM is a crucial feature when studying the interactions between an SCRP and weak MFs. However, the observation of an LFE in a biological system remains challenging due to several factors. In a noisy and spin-rich system such as found in biological systems, different aspects and interactions have to be considered to observe an LFE in the first place. For example, hyperfine interactions between electrons and adjacent nuclear spins become increasingly significant when the external MF becomes weak.^76,77^ These hyperfine interactions will dominate the time evolution of the SCRP states and also lead to a growing number of eigenstates within the spin system. Other interactions, such as inter-radical interactions (the dipolar and exchange interactions), also influence the SCRPs dynamics through the formation of more different eigenstates by lifting the degeneracies between existing eigenstates but are often neglected when theoretically studying the RPM,^47,76,77,154,183^ due to an assumed large separation of the correlated electron spins.

In a steady fluctuating biological environment, several effects perturb the interactions of the SCRP, e.g., the motion of a protein.^76,184,185^ These effects are, similar to RF-MFs, time-dependent fluctuations of the internal interactions experienced by the SCRP, which lead to spin relaxation. Spin relaxation causes the decoherence of an SCRP, eventually suppressing a possible MFE or LFE.^76^ At weak MFs (≪1 mT), both the inclusion of all interspin interactions and their time-dependent changes are of significant importance, as they direct the transitions between the singlet and triplet states of the SCRP and lead to decoherence that diminishes a possible LFE.

Another crucial factor for observing an LFE in the presence of weak static MFs (≤1 mT), which is connected to the decoherence time of the SCRP, is the general lifetime of the SCRP. On the one hand, the SCRP should have a sufficiently long lifetime to allow for slow precession of the electron spins around the external weak MF, thus altering the populations of the singlet and triplet states.^47,54^ On the other hand, an excessively long lifetime leads to decoherence of the SCRP due to spin relaxation effects. Such decoherence ultimately compromises the reaction selectivity of the SCRP, leading to thermally equilibrated spin-state populations and the suppressing of an LFE. The difference between the LFE and the “normal” MFE emerges in a different phase of altering a spin-selective reaction yield of a SCRP resulting in a maximum or minimum at a specific MF flux density.^46^ Figure 6 shows the impact of an increasing external static MF on the triplet state reaction yield ϕ_T_ for a singlet-born SCRP with different lifetimes.^47^ In this example, the SCRP was modeled as a small toy model including three nuclear spins and an external MF.^47^ Spin relaxation and decoherence were considered using a phenomenological approach, as described by Bagryansky et al.^186^

**Figure 6:**
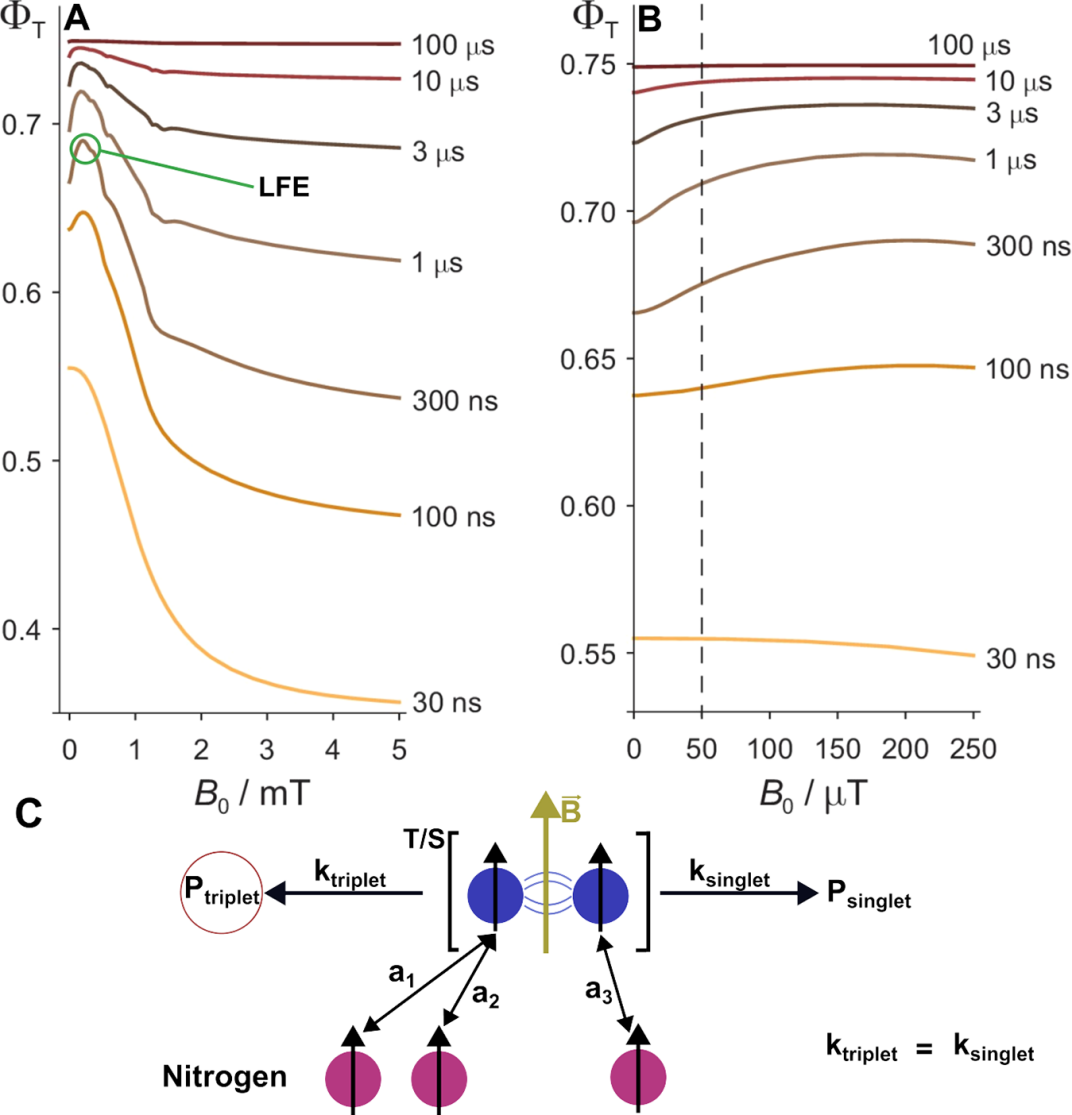
(A) Triplet state reaction yields ϕ_T_ of a toy model SCRP. The SCRP interacts with three nuclear spins and has different lifetimes. An external static MF with a strength ranging from 0 to 5 mT is applied. As can be observed, an LFE at low MFs is only observable at certain lifetimes (maxima of ϕ_T_ in the plots). If the lifetime of the SCRP is too short (30 ns), no LFE (no maxima) can be found, and only the “normal” MFE is observable. If the lifetime of the SCRP is too long (>3 μs) the overall MFE diminishes due to spin relaxation. (B) Zoom in to lower fields, revealing the flattening for very long and short lifetimes. Adapted from from ref 47. CC BY 4.0. (C) Schematic representation of the used SCRP born in a singlet state (S) with equal reaction rate constants for singlet and triplet products. One radical is interacting with two nitrogen spins via isotropic hyperfine coupling constants a_1_ and a_2_, while the other is coupled to only one nitrogen spin via a_3_.

As can be observed, lifetimes exceeding 3 μs reveal a minimal overall MFE over the applied magnetic field strength range (Figure 6B, top two lines) and the triplet state population is near thermal equilibrium (ϕ_T_ = 0.75, all triplet states are populated with 0.25). As previously mentioned, spin relaxation effects significantly alter the spin system populations, inducing increased decoherence as the lifetime extends and thus diminishing any form of MFE. Therefore, not just the SCRP’s overall lifetime matters; the coherence lifetime is equally important.^73^ Reducing the lifetime of the SCRP reveals a peak of ϕ_T_ for *B*_0_ < 1 mT. Notably, a biphasic behavior (with a maximum at low, nonzero *B*) of the triplet yield curve is observed at lifetimes of 3 μs to 100 ns, as illustrated in Figure 6B,^47^ which characterizes the LFE and is well-documented in experimental studies of dyad systems and proteins.^46,154,155,187,188^ When the lifetime of the SCRP is significantly short (∼30 ns) the curve exhibits monophasic behavior with a rapid decrease in triplet-state reaction yield when increasing the MF strength. Here, only the “normal” MFE is found while the LFE does not occur due to the short lifetime of the SCRP. A theoretical understanding of the emergence of an LFE is provided by Lewis et al.^154^ Their study employed a detailed decomposition of the probability amplitudes for the four SCRP states |S⟩, |T_0_⟩, |T_+_⟩, and |T_–_⟩ with and without the influence of several hyperfine interactions. An explanation for emerging peaks at specific low MF strength, as illustrated by Hore’s toy model^47^ in Figure 6, is found as a competitive effect between |S⟩ → |T_±_⟩ interconversion and |S⟩ → |T_0_⟩ interconversion mechanism.^154^ When slowly increasing the strength of the applied static MF, an increase of |S⟩ → |T_0_⟩ interconversion dominates the MFE. In contrast, in higher fields, the steady decrease in the |S⟩ → |T_±_⟩ interconversion is the dominant factor. The LFE arises as an intermediate scenario in which both mechanisms become important.^154^

It becomes apparent that the RPM-based MFE is well-studied for toy model SCRP systems and the influence of static MFs. Different properties, such as several spin–spin interactions, radical motion, diffusion, the lifetime of the SCRP and the coherence lifetime combine in a complex synergy to create an observable MFE and LFE. The theoretical description of these three effects is nontrivial but is mandatory to deliver a framework capable of capturing all properties in the dynamics of an SCRP. The situation becomes even more complex when the SCRP, as found in biological systems, experiences several spin–spin interactions, which are constantly perturbed by thermal effects within the system, and when, additionally, time-dependent external MFs such as RF-MFs are applied.

In the next sections, a detailed overview of spin interactions, equations of motion, and time-dependent effects, which are important for an accurate description of a biologically relevant SCRP, will be provided, serving as a foundation for the reader to delve deeper into the complexity of the RPM and the possible effects of weak RF-MFs on the underlying spin dynamics.

## 10 Magnetic Field Effects in Chemical Systems

One of the main reasons why an RPM for the explanation of magnetic field effects is so attractive is its verification through chemical systems that form stable spin-correlated radical pairs that can be studied systematically. In recent years, many molecular systems^161–163,189,190^ have been synthesized, and magnetic field effects such as the low-field effect^191^ were directly detected.

A prominent example is the observation of magnetically altered reaction yield (MARY) spectra, where the yield of a particular product is tracked with respect to different magnetic field intensities (similar to Figure 6). These MARY spectra are direct evidence that an RPM directs the fate of chemical systems that inherit an SCRP.

Additionally, it was possible to study potential RF-MF effects on molecular systems under controlled conditions, verifying that specific frequency can, in fact, alter chemical reaction yields. Already proposed in the late 20th and early 21st centuries, it was assumed that RF-MF can affect the dynamics of an SCRP through resonance conditions which directly correlate with the spin–spin interactions an SCRP inherits.^191,192^ Through this, a magnetic resonance method, called reaction yield detected magnetic resonance (RYDMR), was developed to study the internal magnetic structure of SCRPs and directly extract, e.g., hyperfine couplings.^193,194^

Jackson et al.^195^ investigated the photochemical reaction of pyrene with 1,3-dicyanobenzene (1,3-DCB) under controlled static magnetic field conditions RF fields with a power of 5 W in the range of 22–25 MHz (SMF and RF-MF). The study demonstrated a significant suppression of the exciplex fluorescence yield at 25 MHz, directly correlating with the hyperfine splitting in the radical pair. This was one of the earliest studies to provide experimental evidence for the resonance condition of RF fields modulating singlet–triplet interconversion in radical pairs. Detailed measurements confirmed the interaction strength between hyperfine couplings and RF-MFs, specifically emphasizing the role of dominant 1,3-DCB hyperfine couplings.

Later Woodward et al.^191^ demonstrated how RF-MFs modulate exciplex fluorescence in anthracene-*d*_10_ and 1,3-dicyanobenzene systems using RF-MFs with frequencies of 34–36 MHz (2, 6, and 10 W) in the presence of the geomagnetic field (RF-MF). Attenuation was observed in the fluorescence signal between 34 and 36 MHz, aligning with the largest hyperfine coupling constants in the 1,3-DCB radical anion. By performing a similar experiment on 1,2-DCB and 1,4-DCB, they were able to support their hypothesis that resonance between the RF-MF and the hyperfine sublevels of the SCRP is required for RF-MFs to alter chemical reaction yields. These observations further solidified the theoretical predictions of the study that singlet–triplet mixing is sensitive to hyperfine-driven RF resonance conditions (0.05 mT, 20–60 MHz field).

Stass et al.^196^ expanded on earlier studies by systematically analyzing the oscillating magnetic field effect (OMFE) in anthracene-d_10_ and 1,3-dicyanobenzene radical pairs under RF-MFs of 1–80 MHz and roughly 0.5 mT (RF-MF). The experiments, carried out in cyclohexanol and acetonitrile mixtures, showed a clear resonance at 36 MHz corresponding to a hyperfine coupling of 0.829 mT in 1,3-DCB. The singlet yield reduction reached 10%, illustrating the impact of both hyperfine interactions and RF amplitude. Simulations based on the RPM provided additional insight into the reaction mechanism under varying RF field strengths.

Woodward et al.^192^ focused on the pyrene and *N*,*N*-dimethylaniline (DMA) radical pair under RF fields spanning 1–80 MHz (∼100 μT, RF-MF). By employing isotopically substituted DMA, distinct resonance features near 46 MHz were attributed to hyperfine couplings with ^14^N. The study highlighted how isotope effects influence radical pair dynamics and reaction yields in the presence of RF fields, providing a more nuanced understanding of singlet–triplet mixing and recombination.

Henbest et al.^197^ presented a diagnostic test for the RPM by examining the recombination of chrysene-d_12_ and 1,4-dicyanobenzene radicals under weak 300 μT RF-MFs. The study explored recombination yields under parallel and perpendicular RF/static field alignments (RF-MF and SMF). A dominant resonance peak emerged at 5 MHz, corresponding to Zeeman splitting comparable to hyperfine interactions, and simulation results highlighted the interplay of RF frequency and field geometry on radical recombination dynamics.

The earlier studies clearly demonstrate that alteration of chemical reactions through external magnetic fields and even radiofrequency fields with intensities in the microtesla to millitesla range can be mediated through an RPM in molecular systems. Albeit the significant progress that was achieved for molecular chemical systems, the picture, however, changes for biological systems in which considerably more spin–spin interactions are to be expected, and the fluctuating environment directly impacts the coherence time of any formed SCRP significantly.

While for the chemical systems, theoretical approaches using simple spin systems such as a three-spin system are often a viable approach to understand the underlying spin dynamics, in a biological system these models are often not applicable. Especially the context of spin relaxation plays a crucial role which is often overlooked when claimed biological effects are explained by simple spin dynamics models.

## 11 Theoretical Framework of the RPM

The following overview serves a dual purpose: first, to equip the reader with crucial knowledge encompassing critical aspects of inherent mechanisms, which are often overlooked during discussions of SCRP spin dynamics simulations, and second, to serve as a foundational summary that is urgently required for future investigations.

Initially, a comprehensive examination of general spin dynamics will be undertaken, followed by an exploration of crucial spin interactions. Subsequently, spin-selective reactions will be contextualized within theoretical frameworks, and a detailed discussion of spin relaxation and time-dependent interactions will be provided.

### 11.1 Quantum Spin Dynamics and Liouville–von Neumann Equation

Dynamical processes of correlated spins require consideration of quantum-mechanical properties. The spin of a particle can be described as a vector in a complex Hilbert space H with dimensions depending on the accessible spin states, which are specified by magnetic quantum numbers of the constituting spins. Systems of *n* spin particles are described by a *n*-fold tensor product (also Kronecker product, shown as ⊗) of the individual spin spaces. An SCRP has a Hilbert space dimension of 4 (|S⟩, |T_+_⟩, |T_0_⟩, and |T_–_⟩); however, through the coupling with *K* nuclear spins in a biological environment, the Hilbert space dimensionality increases drastically. The Hilbert space H of an SCRP is thus

$$H=H_{1}⊗H_{2}{⊗}_{k=1}^{K}H_{k}$$ 1

where $H_{1}$ and $H_{2}$ are the Hilbert spaces of the two electron spins which are coupled to *K* nuclear spins with their respective Hilbert spaces $H_{k}$ . The dynamic description of spin systems and spin-dependent chemical reactions within biological systems is usually accomplished through the density matrix formalism. The advantage of this formalism is that important features, such as mixed state ensembles and spin relaxation, can be accommodated in the equation of motion. The underlying equation of motion for the density matrix formalism is the Liouville–von Neumann (LvN) equation:^87,184^

$$\frac{d}{dt}\mathrm{\rho ̂}\left(t\right)=-i\left[Ĥ\left(t\right),\mathrm{\rho ̂}\left(t\right)\right]-\left\{\mathrm{K̂}\left(t\right),\mathrm{\rho ̂}\left(t\right)\right\}$$ 2

where, ρ̂(*t*) is the time-dependent density matrix of the spin system, *Ĥ*(*t*) is the spin Hamilton operator that includes all important spin interactions, *K̂*(*t*) is the reaction operator describing spin-selective and nonselective chemical reactions. Here and in the following, ℏ = 1. The terms [*Â*, *B̂*] and {*Â*, *B̂*} denote the commutator and anticommutator of the operators *Â* and *B̂*, respectively. The spin Hamiltonian and the density matrix are operators acting within the Hilbert space H , derived as projections onto subspaces that preserve the same dimensionality.

For spin-selective reactions, the quantum yields Φ_Θ_ (Θ = |S⟩, |T_0_⟩, |T_+_⟩, |T_–_⟩) of a specific state reaction are commonly given by^184,198^

$$\Phi_{\Theta }=\int_{0}^{\infty }k_{\Theta }\left(t\right)P_{\Theta }\left(t\right)\;dt$$ 3

with the reaction rate *k*_Θ_(*t*) and time-dependent population of the spin state of interest *P*_Θ_(*t*) obtained using

PΘ(t)=Tr[P̂Θ ρ̂(t)] 4

where *P̂*_Θ_ is the projection operator of a given state Θ, Tr is the trace of the Hilbert space, and ρ̂(*t*) is the time-dependent density matrix obtained by propagating the density matrix ρ̂(0) at *t* = 0 with the time-evolution operator *Û*(*t*, 0):^199^

$$\mathrm{\rho ̂}\left(t\right)=Û\left(t,0\right)\mathrm{\rho ̂}\left(0\right)Û{\left(t,0\right)}^{†}$$ 5

Equation 5 is a solution to eq 2 when defining the time-evolution propagator *Û*(*t*_1_, *t*_0_) with *t*_1_ = *t*_0_ + δ*t*, which evolves the state from *t*_0_ to *t*_1_ as^198^

$$Û\left(t_{1},t_{0}\right)=\mathrm{T̂}\;\exp\left[\int_{t_{0}}^{t_{1}}d\tau \;\left(-iĤ\left(\tau \right)-\mathrm{K̂}\left(\tau \right)\right)\right]$$ 6

where *T̂* is the time-ordering operator.

In the context of the RPM, the initial condition of the density matrix ρ̂(0) is represented as the tensorial product of the projector on the initial state |Θ_init_⟩ and the identity operator $I_{Z}$ of the nuclear spins with Hilbert subspace size *Z* (assuming one initial electron spin state):

$$\mathrm{\rho ̂}\left(0\right)=\frac{1}{Z}\left(\left|\Theta_{\mathrm{init}}\right\rangle \langle \Theta_{\mathrm{init}}|\right)⊗I_{Z}$$ 7

For an SCRP, |Θ_init_⟩ is the initial spin state of the electron spins. In biological systems, such as proteins, many nuclear spins must be considered in the spin dynamics, leading to numerous nuclear spin states in $I_{Z}$ and consequently to the exponential growth of the Hilbert space due to the *K*-fold Kronecker product in eq 1.

A commonly used approach to straightforwardly solve the LvN equation when the observable of interest has to be evaluated from *t* = 0 → *t* = *∞*, the spin Hamiltonian is time-independent, and dissipative effects have to be considered is the transformation into the Liouville space or superoperator space.^48^ In the case of the dynamics of a biologically relevant SCRP, the overall quantum yield for a decaying SCRP is required which becomes resource intensive when propagating the spin system on a numerical time grid stepwise. In the Liouville space, the density matrix is transformed into a vector of *n* ^2^ × 1 length, while the acting operators are expanded to dimension *n* ^2^ × *n* ^2^ (where *n* is the size of the full H ):

[Ĥ,ρ̂]=Ĥρ̂−ρ̂Ĥ→(I⊗Ĥ−ĤT⊗I)ρ⃗^→H^^ρ⃗^ 8

where in the case of the inclusion of a reaction operator −i*Ĥ* – *K̂* the overall superoperator is denoted as L^^ (^T^ indicates the transpose). While expanding the dimensionality of the Hamiltonian through the transformation into Liouville space, two advantages appear. First, the commutators that appear in eq 2 are accomplished by simple multiplication which becomes further advantageous when more complex commutator structures are introduced into the equation of motion via relaxation operator terms as will be discussed later. Second, the calculation of the quantum yield Φ_Θ_ from *t* = 0 → *t* = *∞* simplifies using the Laplace transform under the assumption that the SCRP are fully decayed when *t* →*∞*. The following rule emerging in the Laplace transform L may be used to transform eq 3:^48^

$$L\left\{\frac{d}{dt}\mathrm{\rho ̂}\left(t\right)\right\}\left(s\right)=sL\left\{\mathrm{\rho ̂}\right\}\left(s\right)-\mathrm{\rho ̂}\left(0\right)$$

$$\underset{s\to 0}{\lim}sL\left\{\mathrm{\rho ̂}\right\}\left(s\right)=\underset{t\to \infty }{\lim}\mathrm{\rho ̂}\left(t\right)$$ 9

Here, the first equation can be used to perform the following transformation:

ddtρ̂(t)=L^^ρ⃗^⁣→⁣sL{ρ̂}(s)−ρ̂(0)=L^^L{ρ̂}(s) 10

By rearranging to solve for L{ρ̂}(s) one obtains

L{ρ̂}(s)=(sI−L^^)−1ρ̂(0) 11

where I is the identity. By assuming *s* → 0 and substituting into eq 3 one derives:

ΦΘ=kΘTr(P̂Θ∫0∞ρ̂(t)dt)=kΘTr(P̂ΘL{ρ̂}(0))=−kΘTr(L^^−1ρ⃗^(0)) 12

which allows for avoiding the propagation of the density matrix over a grid of several time steps and directly computing the reaction quantum yield after infinite time. However, the transformation is only viable for time-independent superoperators L^^ because these superoperators are exchanged with the Laplace transform in eq 10. There are approaches and modifications to a time-dependent Hamiltonian to allow for the usage of eq 12 which will be discussed in later sections. The next section, however, will first discuss the construction of the spin Hamiltonian *Ĥ* with all relevant interactions and will provide detailed aspects and features of each interaction concerning an SCRP in a biological system.

### 11.2 Spin Interactions

In the following, a discussion of all important spin interactions within an SCRP in biological systems is provided. Besides the mathematical formulation of each interaction, comments concerning the RPM are included to lay a foundation for the theoretical understanding of possible RF-MF effects on an SCRP.

#### 11.2.1 Zeeman Interaction

One of the most crucial interactions for the emergence of an MFE through the RPM is the interaction of an SCRP with an external MF, called the Zeeman interaction. The Zeeman interaction *Ĥ* ^Zn^ is defined as^200,201^

ĤiZn=μBB⃗·gi·S⃗^i 13

where *g*_*i*_ is the *g* matrix, a 3 × 3 matrix describing the magnetic moment of the electron in a radical and the coupling strength of spin S⃗^i = [*Ŝ*_*ix*_, *Ŝ*_*iy*_, *Ŝ*_*iz*_] with an MF vector *B⃗*. *Ŝ*_*ik*_ are the Cartesian spin operators constructed through Pauli matrices. The constant μ_B_ is the Bohr magneton. In a molecule, the nuclear spins are also experiencing the force of an external MF; however, the interaction strength in comparison to an electron spin is magnitudes smaller and is, thus, often neglected at low MF strengths.^46,202^ Considering RF-MFs, the time-dependent oscillations of the field lead to a time-dependency of the Zeeman interaction itself. Thus, if the applied MF is a sum of a static MF *B⃗*_0_ and an oscillating component *B⃗*_1_(*t*) at a specific frequency ν_*rf*_, transitions between eigenstates of the SCRP system may be induced depending on the spin system energy eigenvalue differences.

#### 11.2.2 Hyperfine Interaction

If the MF strengths in the Zeeman term are weak, such as for the geomagnetic field or anthropogenic RF-MFs, other interactions, such as the hyperfine interaction, become more dominant for the time evolution of an SCRP.^44,76,77,184^ In a molecular environment, the electron spins are located close to nearby nuclei, which also possess a magnetic moment and, thus, induce MFs that interact with the electron spins in the form of a hyperfine interaction. Figure 7 illustrates the regime of importance for the hyperfine interaction concerning the external MF strength for a reaction yield of an SCRP. The hyperfine interaction of an electron spin with a nuclear spin can be formulated as^44,46^

ĤikHf=S⃗^i·Aik·I⃗^k 14

where *A*_*ik*_ is the 3 × 3 coupling matrix describing the coupling strength between the electron spin *i* and the nuclear spin I⃗^k . The coupling matrix *A*_*ik*_, which is predominantly constructed from two contributions (the isotropic Fermi contact interaction *A* ^iso^ and anisotropic dipole–dipole interaction *A* ^aniso^), is directly connected to the spin density of a system and the spin density distance to a certain nucleus (ℏ = 1):^203^

$$A^{\mathrm{iso}}=\frac{2\mu_{0}g_{e}\mu_{B}g_{n}\mu_{n}}{3}|\Psi_{0}\left(0\right){|}^{2}$$ 15

$$A^{\mathrm{aniso}}=\frac{\mu_{0}g_{e}\mu_{B}g_{n}\mu_{n}}{4\pi }\langle \Psi_{0}\left|\frac{3r_{q}r_{p}-\delta_{qp}r^{2}}{r^{5}}\right|\Psi_{0}\rangle$$ 16

where *g*_e_ is the *g* value of the electron, *g*_n_ is the *g* value of the nucleus, μ_0_ is the vacuum magnetic permeability, μ_n_ is the magnetic moment of the nucleus, |Ψ_0_(0)|^2^ is the ground-state probability spin density of the electron at the site of the nucleus, |Ψ_0_⟩ is the ground-state wavefunction, *r* is the distance between the electron density and the nucleus, and *r*_*q*,*p*_ are the respective Cartesian components of the distance *r⃗* between the nucleus and the electron spin. In the case of freely and rapidly tumbling radicals, the anisotropic part of the hyperfine interaction can be ignored because its contributions are averaged to zero.^204^ For SCRPs within protein structures, however, the anisotropic parts become relevant due to the rotational motion being slow or comparable to the spin dynamics.^77,184^ Anisotropic hyperfine interactions are particularly important in, e.g., providing directional magnetic field effects, which is an essential requirement for a compass sense and possibly other forms of magnetoreception.^55^ In a freely tumbling radical, anisotropic contributions average out, leaving no directional preference. However, when a radical pair is constrained within a protein or embedded in a membrane environment, its orientation relative to the external magnetic field becomes fixed or slowly varying. This geometrical restriction prevents the anisotropic hyperfine terms from averaging, thereby introducing orientation-dependent spin dynamics. As a result, the potential spin-selective reaction yield of an SCRP may become sensitive to the angle between the molecular frame and the external magnetic field.

**Figure 7:**
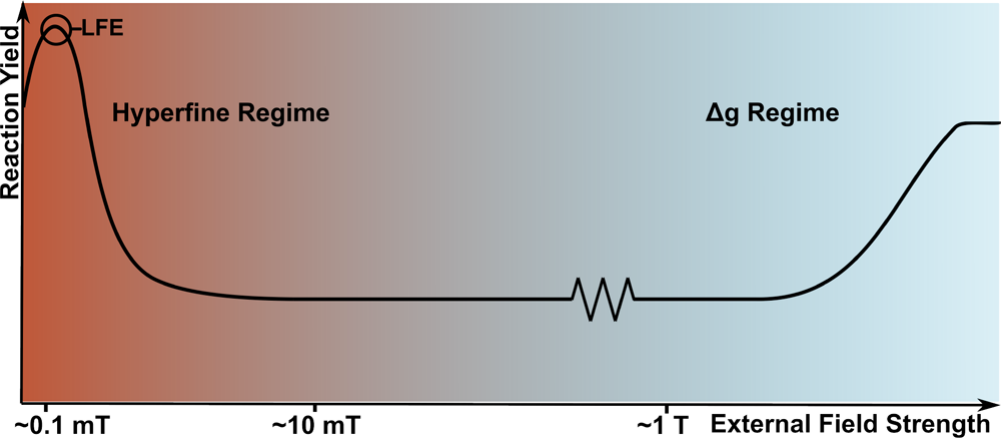
External MF regimes and their dominating effects. At weak static MFs, where the LFE occurs, hyperfine interactions dominate the dynamics of an SCRP. At high MF strengths, other mechanisms, such as the Δ*g* mechanism, become dominant. A similar description of magnetic field effects can be found in ref 44.

Such anisotropy is believed to be crucial in magnetoreception. For example, cryptochrome proteins, which are candidate magnetosensors, are thought to be linked to the membranes of retinal double cone cells in birds.^55,184,205^ This binding to a structured environment introduces anisotropy into the hyperfine interactions and enables directional information to be encoded in the spin-selective reaction yields.^54^ The degree of anisotropy in hyperfine couplings thus directly impacts the compass sensitivity, and structural features such as planar arrangements or preferential alignment of radical-bearing domains could tune the magnetic response.

Furthermore, the constant motions of the radical molecules lead to complex time-dependency of the hyperfine interactions inducing spin relaxation effects on an SCRP.^76,184^ When weak oscillating MFs, such as RF-MFs, perturb the SCRP spin system, the hyperfine interactions most likely dominate the spin dynamics. Through the comparably strong coupling (compared to the weak RF-MFs) of the electron spin with nuclear spins, several possible eigenstates are formed, which are populated. The growing number of eigenstates leads to increased possible transitions and interconversion between different spin states, which may be triggered by external perturbations, such as RF-MF (Figure 8).

**Figure 8:**
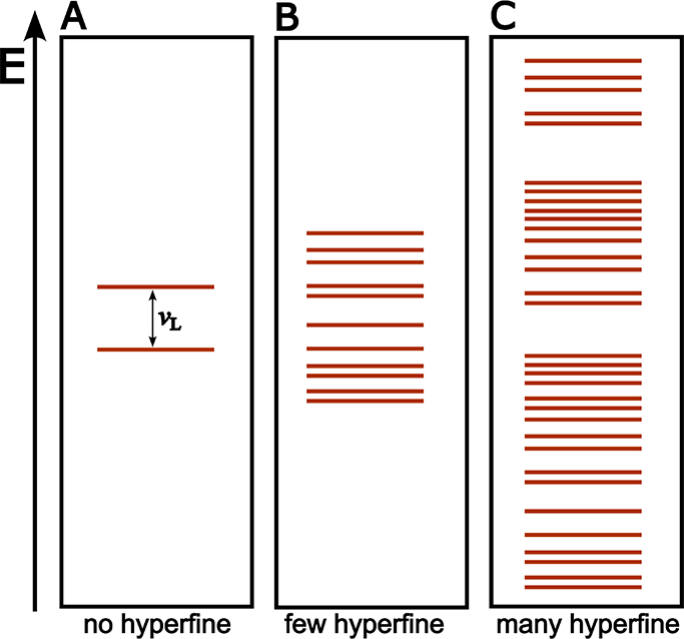
Schematic representation of the eigenstates of an electron spin when (A) no other interactions than the external magnetic field are considered, (B) a few hyperfine interactions are considered, and (C) several hyperfine interactions are included. The frequency difference between the two states in (A) characterizes the MF-dependent Larmor frequency (ν_L_ = 1.316 MHz in the geomagnetic field) of an electron spin that is not interacting with other spins.

The formation of a discrete set of eigenstates implies that, in general, not all frequencies of RF-MFs will affect the SCRP if the frequency surpasses energy differences above the highest energy difference between the eigenstates.^56^ The consideration of the SCRP coupling with nuclear spins is thus of significant importance when studying the spin dynamics under the influence of weak RF-MFs.

#### 11.2.3 Dipolar Interaction and Exchange Interaction

Similar to the hyperfine interaction, where an electron spin interacts with a nuclear spin, both spins within an SCRP induce an MF, which is felt by the respective counterpart as a dipolar coupling. Dipolar coupling significantly impacts the dynamics of a SCRP when the external MF is weak, and the distance between the two correlated electron spins is short. The coupling strength drops off as the inverse cube of the distance between the two electron spins. For instance, when the SCRP is interacting with the geomagnetic field which has a strength of ∼50 μT, the dipolar coupling within the SCRP has a significant influence on the spin dynamics within ∼3.8 nm (the dipolar coupling strength equals ∼50 μT at this distance).^46,87,206^ The dipolar interaction can be described in terms of spin operators similar to the dipole–dipole part of the hyperfine interactions as^203^

ĤijD=S⃗^i·Dij·S⃗^j 17

where *D*_*ij*_ is the (3 × 3) dipolar coupling tensor, which in the point dipole approximation assumes the form (ℏ = 1)

ĤijD=−μ0μB2gigj4πr3[S⃗^i·S⃗^j−3r2(S⃗^i·r⃗)(S⃗^j·r⃗)] 18

The electron–electron contact (similar to the Fermi contact for the hyperfine interactions) is commonly ignored due to the large distance between the two radicals. For small free radicals and fast diffusion, the diffusive motion of one radical around the other causes the dipolar interaction to average out to zero. In low-field situations, however, the averaging may become more complicated. The dipolar interaction frequently inhibits the interconversion of states within an SCRP, thereby counteracting MFEs in weak magnetic fields. Numerous studies have demonstrated that the introduction of dipolar interaction leads to a substantial diminishment of MFEs.^46,75,207^ For instance, in the case of magnetoreception, the dipolar interaction decreases the external MF angle dependency (anisotropy plots^76^) of the SCRP quantum yield ratio significantly, casting doubt on the validity of the naive RPM as a possible mechanism for a magnetic compass of migratory songbirds.^75,206,207^

Another purely quantum-mechanical intrinsic spin interaction of an SCRP is the exchange between the two spins. The exchange interaction arises through the indistinguishability of correlated quantum particles in combination with the Pauli exclusion principle. However, the effective distance of this interaction is short^206^ and is, thus, often neglected. The exchange interactions can be formulated as

Ĥijex=−2JS⃗^i·S⃗^j 19

where *J* is the exchange coupling constant which is half the energy difference of the electronic structure between the triplet and the singlet states. The exchange coupling within an SCRP becomes important when the radicals are close, leading to an altering of the MFE. Within spin dynamics, it is often assumed that the interaction strength decreases exponentially with the SCRP separation distance:^198^

$$J\left(r\right)\approx J_{0}\cdot e^{-\beta r}$$ 20

where *J*_0_ is the exchange at *r* = 0, *r* is the inter-radical distance, and β is a range parameter.^198,207^

When the exchange coupling *J* is comparable with, or smaller than, the hyperfine couplings, singlet–triplet interconversion takes place and can be influenced by applied MFs. If *J* is much larger than the hyperfine couplings, however, an SCRP will be locked into its initial state unless the electron Larmor frequency more or less matches 2*J* ^46^ (so-called *J* resonance). In principle, both radical–radical couplings (dipolar and exchange) will decrease a possible MFE due to the suppression of singlet–triplet interconversion through the lifting of zero-field degenerate states.^207^ Characteristically *D* and *J* depend on inter-radical distance *r* as 1/*r* ^3^ and e^–β*r*^, respectively. The slower decay of *D* implies that for larger distance it is often the more important inter-radical interaction. It was demonstrated by Efimova and Hore, however, that the dipolar and exchange coupling may compensate each other when the following condition is met:^206,207^

$$J=\left(\frac{3}{4}q^{2}-\frac{1}{2}\right)D$$ 21

Here, *q* ∈{0, ±1}.^207^ The effect is known as *J*/*D* cancellation. Nevertheless, Hiscock et al.^75^ and Babcock et al.^207^ demonstrated that including dipolar interaction suppresses the magnetic field effects within the SCRP of cryptochrome drastically, leading to a challenge for the RPM hypothesis within magnetoreception. A recent study by Smith et al., however, illustrated that the time-dependencies of intra-SCRP interactions such as the exchange may have a significant impact in increasing the effectiveness of the magnetic compass when the interaction has specific frequencies.^198^

The intra-SCRP interactions also play a crucial role in magnetic resonance methods such as photo-CIDNP (chemically induced dynamic nuclear polarization), where detected polarization of nuclear spins can be explained, for example, by the so-called three spin mixing (TSM).^87^ The TSM combines the hyperfine interactions between the nuclear and one of the SCRP radicals with the intra-SCRP electron spin–spin interactions, resulting in significant intensity increases or decreases of peaks in an NMR spectrum. Using photo-CIDNP and explaining the observed intensities through the TSM supported the existence of SCRP and the functioning of the RPM in several biological systems such as the light-oxygen-voltage (LOV) domain protein or the photosynthetic reaction center of the bacterium *Rhodobacter sphaeroides*.^87,208^ Furthermore, in chemically induced dynamic nuclear polarization (CIDEP), the exchange interaction plays a crucial role in producing polarization and the technique can be employed to directly measure the potential influence of electron–electron spin interactions within a biological system.^209,210^ Thus, the dipolar and exchange interactions provide an important contribution to the spin dynamics of an SCRP in a biological system, which will remain and become even more dominant under the influence of weak magnetic fields. Even though MFEs of weak magnetic fields are often diminished by intra-SCRP interactions, complex time-dependencies of these interactions might reveal an enhancement of the MFE^198^ and must be additionally considered in noisy environments, such as biological systems.

#### 11.2.4 Spin–Orbit Coupling

Spin–orbit coupling (SOC) becomes crucial for radicals that undergo constant and rapid rotation or molecules exhibiting high orbital angular momenta (i.e., transition-metal complexes).^125,211^ As an example, the superoxide ion (O_2_^•–^), which is often mentioned in the context of an RPM-based MFE,^10,69,116–118^ showcases a prominent SOC interaction, leading to notable spin relaxation. The induced spin relaxation can, in turn, suppress an MFE between O_2_^•–^ and X^•^ within a [O_2_^•–^–X^•^] SCRP,^125,151^ where X^•^ is an arbitrary radical that experiences several hyperfine interactions.

At its core, SOC originates from the interaction of an electron’s spin with its associated orbital angular momentum. In situations where the radical of interest rotates rapidly, possessing an orbitally degenerate electronic ground state and strong SOC, the quantization of spin angular momentum along its molecular axis may cause decoherence effects in an SCRP. Such a phenomenon can primarily be found for the spin of O_2_^•–^.^125^ The SOC spin Hamiltonian is expressed as^125^

ĤijSO=λijS⃗^i·L⃗^j 22

where, λ_*ij*_ denotes the SOC strength and L⃗^j represents the electron’s orbital angular momentum.

Several experiments have attributed the generation of ROS to superoxide.^9,10,212^ Given these findings, it is paramount to incorporate the effects of SOC to accurately depict the spin dynamics of possible SCRPs within biological systems. The SOC presents one of the most crucial spin interactions for rapidly rotating radicals, and neglecting SOC is only viable if an otherwise rapidly rotating radical is immobilized, e.g., through binding in a protein pocket.^116^ Once SOC effects get suppressed—either by scavenging of superoxide or by asymmetric environments eliminating orbital degeneracy—the O_2_^•–^ species may become detectable through EPR measurements at room temperature.^125,213^ However, EPR spectroscopy has only detected superoxide-containing SCRPs in static MFs much stronger than the geomagnetic field, approximately 50 μT.^125^

### 11.3 Spin-Selective Reactions

Spin-selective reactions are one of the key aspects for the observation of an RPM-based MFE. In biological systems, where magnetic field effects are claimed, e.g., NADPH oxidase,^68^ the spin-selective reactions of interest are often unknown and nontrivial to identify experimentally.

Through the sensitivity in a certain field (e.g., the geomagnetic field in the case of magnetoreception) it is possible to assume a potential bound for the required lifetime of an SCRP.^55,214^ For example, for the interaction with the geomagnetic field of ∼50 μT, the SCRP must at least have a lifetime of 1 μs.^55,214^ Combined molecular dynamics and quantum chemical calculations can also provide reaction rate constants that may be used for spin dynamics calculations.^55,215^ In rare cases, spin-selective reactions are measured via time-resolved absorption spectroscopy.^216^ Spin-selective reactions are commonly assumed to behave as first-order reactions (the reaction rate is linear depending on the concentration of only one reactant), which can be described using an exponential decay model. The spin-selective reactions are expressed within the spin system’s Hamiltonian via reaction operators such as the Haberkorn operator:^184,217,218^

$$\mathrm{K̂}=\underset{\Theta }{\sum }\frac{k_{\Theta }}{2}{\mathrm{P̂}}_{\Theta }$$ 23

where *k*_Θ_(*t*) is the reaction rate constant of a specific spin-selective reaction of state Θ ∈{|S⟩, |T_0_⟩, |T_+_⟩, |T_–_⟩} and *P̂*_Θ_ is the projection operator of state Θ. The operator in eq 23 is embedded into the LvN equation via

$$\frac{d}{dt}\mathrm{\rho ̂}\left(t\right)=-i\left[Ĥ\left(t\right),\mathrm{\rho ̂}\left(t\right)\right]-\left\{\mathrm{K̂},\mathrm{\rho ̂}\left(t\right)\right\}$$ 24

where {*Â*, *B̂*} is the anticommutator between the two operators *Â* and *B̂*. The Haberkorn operator was recently derived by a perturbative treatment by Fay et al.^219^ and is commonly applied in the spin chemistry community.^76,77,184^ However, other descriptions exist, such as the Jones–Hore reaction operator^218^ and the Kominis operator^220^ for the description of a dissipative loss of the density matrix.

The precise knowledge of all possible spin-selective and spin-independent reactions in an SCRP is fundamentally important for the accurate description of a possible MFE. Not only chemical reactions, but also diffusive processes that lead to the separation of a radical pair play a crucial role in the time evolution of the spin system.^86^ The determination of the reaction rate constant is, however, a tedious process which is still a state-of-the-art challenge in theory and experiment. Lack of knowledge about spin-selective reactions is often one of the most problematic topics when investigating a SCRP in a biological system.

### 11.4 Incorporation of RF-MFs into Spin Dynamics

Understanding the interaction between an SCRP with a specified lifetime and an external MF paves the way for delving into the potential impact of RF-MFs on the SCRP dynamics. Typically, oscillating RF-MFs can be described as a time-dependent oscillation along a certain Cartesian direction, which may be expressed as follows:^74,221,222^

ĤRFC(t)=μBB1(cos(ωt+γ)sin(ωt+γ)0)·gi·S⃗^i 25

ĤRFL(t)=μBB1(00cos(ωt+γ))·gi·S⃗^i 26

where *B*_1_ is the amplitude of the MF, ω is the frequency, and γ is the phase shift. Here, *Ĥ*_RF_C__(*t*) describes circularly polarized RF-MFs in the *xy* plane, while *Ĥ*_RF_L__(*t*) describes linearly polarized RF-MFs along the *z* axis. Central to RF-MFs are the frequency ω and the amplitude *B*_1_, both of which affect the eigenstates of the SCRP within a single period. For weak anthropogenic RF-MF effects, broadband RF-MF is a more realistic approach to study the alteration of the SCRP dynamics through external oscillating MFs. Here, a sum of frequencies is commonly employed, and the overall intensity is evaluated by the root-mean-square relation between all considered frequencies as described in previous studies.^4,75^ The frequency domain of anthropogenic RF-MFs spans broadly from megahertz to gigahertz. However, the amplitudes of these fields remain, in general, low (nT to μT), resulting in extremely weak MF strengths. The consideration of RF-MFs introduces time-dependency into the Hamiltonian, which further complicates the equations of motion. Several approaches to incorporate complex time-dependent effects in spin dynamics calculations of SCRPs were employed in the last decades. The description of these theories and their advantages and disadvantages will be described in the next section.

### 11.5 Theoretical Description of Complex Time Dependencies

In biological systems, interactions of an SCRP (e.g., with adjacent nuclear spins) are constantly perturbed, altering the populations and coherences of SCRP spin states toward thermal equilibrium.^76,184^ As stated by several authors,^47,73,76^ not only is the SCRP lifetime itself of major importance, but also the decoherence time of an SCRP to observe an MFE. RF-MFs, while having a small amplitude, also emerge as time-dependent fluctuations within the Hamiltonian of the SCRP system. Both the RF-MFs and the molecular motion-induced time-dependency of internal spin interactions lead to significant effects, such as spin relaxation drastically changing the spin dynamics of an SCRP compared to a nonperturbed SCRP. Furthermore, time-dependent magnetic fields could be engineered to exert control over reactions involving radical pair intermediates using methods of optimal control.^223–227^

The induced perturbations and subsequent altering of the populations of spin states may have positive or negative effects in a biological environment by either diminishing or enhancing the reaction yields of certain spin-selective chemical reactions. For example, in the case of magnetoreception, spin relaxation induced due to protein motion suppresses the required anisotropy through fluctuations of hyperfine interactions,^76^ a new study by Smith et al. demonstrates that oscillatory motion may have a positive influence through the induced fluctuations of intraradical interactions.^198^ It is thus crucial to have a sufficient description of complex time-dependencies to investigate possible MFE and subsequent effects through RF-MFs.

The following sections explore and illustrate the most prominent theoretical approaches to incorporate complex time-dependencies in the spin Hamiltonian. Here, the aim is to provide a profound overview for future theoretical investigations of SCRP in biological systems.

#### 11.5.1 Phenomenological Approach: The Lindblad Equation

The Lindblad equation is fundamental in quantum mechanics and is used to describe the time evolution of the density matrix for a quantum system, including both the unitary dynamics and the dissipative dynamics. The latter takes into account relaxation and decoherence due to interaction with an environment that is often referred to as the “bath”.^184,228,229^ The Lindblad equation is an extended version of the previously discussed Liouville–von Neumann equation and provides a general framework that allows studying open quantum system coupled to an environment. In the context of spin systems, it provides a mathematical framework for modeling the dissipative behavior of correlated electron spins in a fluctuating environment, which is essential for understanding the dynamics of RPs in biological systems.^73,76,214,230^

The general form of the Lindblad equation for the time evolution of a density matrix ρ̂ is given by^231^

$$\frac{d\mathrm{\rho ̂}}{dt}=-i\left[Ĥ,\mathrm{\rho ̂}\right]+\underset{i}{\sum }k_{i}\left({Ô}_{i}\mathrm{\rho ̂}{Ô}_{i}^{†}-\frac{1}{2}\left\{{Ô}_{i}^{†}{Ô}_{i},\mathrm{\rho ̂}\right\}\right)$$ 27

where *Ô*_*i*_ are system-specific operators that describe the interactions with the environment and *k*_*i*_ is the dephasing rate constant. The Lindblad equation is often used in theoretical investigations when no precise knowledge of the coupling between the quantum system and the environment is required and only phenomenological dephasing effects are important. In terms of the RPM, the Lindblad equation is often used when toy model SCRP systems are investigated that are required to explain fundamental behaviors of the SCRP without considering too much detail.^73^ While the Lindblad formalism excels in phenomenological studies, it may not always be ideal for describing complex relaxation mechanisms due to the often missing or nontrivial to acquire dephasing rate constants, which highlights its primary application in providing valuable insights into simplified systems. The influence of RF-MFs is additionally not straightforwardly describable using the Lindblad approach. Nevertheless, its straightforward mathematical structure allowed for many pioneering studies to explore the behavior of SCRPs.^73,133^

#### 11.5.2 Stochastic Fluctuations

Considering quantum systems, such as an SCRP, in a biological environment leads to the understanding that the explicit description of external perturbations, such as the random motions of a protein consisting of thousands of atoms, is nontrivial. Thus, it is more convenient to evaluate the perturbation of the dynamics of an SCRP through the biological environment stochastically. Here, the key principles are to treat the external perturbation as weak and to condense the complex time-dependent fluctuations into correlation functions, which eventually leads to time-independent equations that can be treated more easily.

Many stochastic master equations have evolved,^228,232^ but only two theories will be discussed in this review due to their frequent usage in spin dynamics and the RPM.

#### 11.5.3 Bloch–Redfield–Wangsness Theory

A popular stochastic perturbative relaxation theory is the Bloch–Redfield–Wangsness (BRW) theory.^76,86,184,185,233–235^ The approach, first derived by Redfield,^236^ delivers a quantum master equation that describes the interaction between a spin system and its environment as a perturbation introduced through stochastic functions. For those seeking a comprehensive and in-depth derivation of the BRW theory, a myriad of textbooks and scholarly articles are available, offering deep insights and explanations.^228,232,235^ However, it is imperative to note that this review is confined to providing a succinct overview of the fundamental formalism integral to the BRW theory without delving into the exhaustive details and complexities.

In its essence, the BRW theory is dedicated to elucidating the interaction mechanisms between a spin system, represented symbolically as S, and its corresponding environment or bath, denoted as B. From a mathematical point of view, the total Hamiltonian, represented as *Ĥ*_tot_, characterizing the spin system and the bath, can be succinctly factorized, as described in the literature:^228^

$${Ĥ}_{\mathrm{tot}}={Ĥ}_{S}+{Ĥ}_{B}+{Ĥ}_{I}\left(t\right)$$ 28

where *Ĥ*_S_ is the static spin system Hamiltonian, *Ĥ*_B_ is the bath Hamiltonian, and *Ĥ*_I_ describes the time-dependent interaction between the bath and the spins.

The primary objective of the BRW theory is to strategically omit the explicit description of the dynamics inherent to the bath, represented by *Ĥ*_B_, and to concentrate predominantly on the dynamics of the spin system. Furthermore, BRW theory aims to modify *Ĥ*_I_ to encapsulate time-dependent interaction phenomena through a time-independent coupling function.

Given the assumption that the ensemble-averaged expectation value of *Ĥ*_I_ is zero and considering that the bath remains unaffected by the dynamics of the system, the evolution of the system, under the influence of a perturbation with amplitude significantly lower than the static Hamiltonian *Ĥ*_S_, can be described up to second order in *Ĥ*_I_ ^184,228^ as

$$\frac{d{\mathrm{\rho ̂}}_{S}\left(t\right)}{dt}=-\int_{0}^{t}dt^{\prime }\;{\mathrm{Tr}}_{B}\left[{Ĥ}_{I}\left(t\right),\left[{Ĥ}_{I}^{†}\left(t^{\prime }\right),{\mathrm{\rho ̂}}_{S}\left(t^{\prime }\right)⊗{\mathrm{\rho ̂}}_{B}\right]\right]$$ 29

where ρ̂_S_(*t*) is the density operator of the spin system, ρ̂_B_ is the density operator of the bath, and Tr_B_ is the trace over the bath states. In the following, all derivations are in the interaction picture, unless otherwise stated. Equation 29 is based on the assumption (often called the Born approximation) that the complete density operator ρ̂(*t*) (system and bath) can be factorized as^228^

$$\mathrm{\rho ̂}\left(t\right)\approx {\mathrm{\rho ̂}}_{S}\left(t\right)⊗{\mathrm{\rho ̂}}_{B}$$ 30

Equation 29 is non-Markovian, as the evolution of the density matrix at a time *t* depends on the previous time instances *t*′ < *t*.

A further simplification of eq 29 postulates Markovian behavior of the spin system (the Markov approximation). First, assuming that ρ̂_S_(*t*) changes slowly with *t*, eq 29 can be rewritten as^228^

$$\frac{d{\mathrm{\rho ̂}}_{S}\left(t\right)}{dt}=-\int_{0}^{t}dt^{\prime }\;{\mathrm{Tr}}_{B}\left[{Ĥ}_{I}\left(t\right),\left[{Ĥ}_{I}^{†}\left(t^{\prime }\right),{\mathrm{\rho ̂}}_{S}\left(t\right)⊗{\mathrm{\rho ̂}}_{B}\right]\right]$$ 31

Although this equation is local in time, it is still non-Markovian due to the time evolution of the density matrix ρ̂_S_(*t*) that depends on the explicit choice of the initial condition ρ̂_S_(0). Second, a Markovian form of eq 31 can be obtained if the substitution *t*′ → *t* – *t*′ is performed and the upper limit of the integral is extended to *∞*:

$$\frac{d{\mathrm{\rho ̂}}_{S}\left(t\right)}{dt}=-\int_{0}^{\infty }dt^{\prime }\;{\mathrm{Tr}}_{B}\left[{Ĥ}_{I}\left(t\right),\left[{Ĥ}_{I}^{†}\left(t-t^{\prime }\right),{\mathrm{\rho ̂}}_{S}\left(t\right)⊗{\mathrm{\rho ̂}}_{B}\right]\right]$$ 32

In general, the interaction operator *Ĥ*_I_ is decomposed into a sum of direct operator products *Ŝ*_α_ describing the spin system and operators *B̂*_α_ describing the bath:

$${Ĥ}_{I}\left(t\right)=\underset{\alpha }{\sum }{Ŝ}_{\alpha }\left(t\right)⊗{\mathrm{B̂}}_{\alpha }\left(t\right)$$ 33

in which *Ŝ*_α_ and *B̂*_α_ can be chosen arbitrarily as long as *Ĥ*_I_(*t*) = *Ĥ*_I_^†^(*t*). A reformulation of eq 32 leads to

$$\frac{d{\mathrm{\rho ̂}}_{S}\left(t\right)}{dt}=-\underset{\alpha ,\beta }{\sum }\int_{0}^{\infty }dt^{\prime }\;g_{\alpha \beta }\left(t^{\prime }\right)\left({Ŝ}_{\alpha }\left(t\right){Ŝ}_{\beta }^{†}\left(t-t^{\prime }\right){\mathrm{\rho ̂}}_{S}\left(t\right)-{Ŝ}_{\beta }^{†}\left(t-t^{\prime }\right){\mathrm{\rho ̂}}_{S}\left(t\right){Ŝ}_{\alpha }\left(t\right)\right)+g_{\alpha \beta }^{*}\left(t^{\prime }\right)\left({\mathrm{\rho ̂}}_{S}\left(t\right){Ŝ}_{\beta }\left(t-t^{\prime }\right){Ŝ}_{\alpha }^{†}\left(t\right)-{Ŝ}_{\alpha }^{†}\left(t\right){\mathrm{\rho ̂}}_{S}\left(t\right){Ŝ}_{\beta }\left(t-t^{\prime }\right)\right)$$ 34

where *g*_αβ_(*t*′) = Tr_B_(*B̂*_α_(*t*)*B̂*_β_^†^(*t* – *t*′)­ρ̂_B_) = ⟨*B̂*_α_(*t*)*B̂*_β_^†^(*t* – *t*′)⟩ is the bath correlation function. Usually *g*_αβ_(*t*′) are homogeneous in time:

$$\left\langle {\mathrm{B̂}}_{\alpha }\left(t\right){\mathrm{B̂}}_{\beta }^{†}\left(t-t^{\prime }\right)\right\rangle =\left\langle {\mathrm{B̂}}_{\alpha }\left(t^{\prime }\right){\mathrm{B̂}}_{\beta }^{†}\left(0\right)\right\rangle$$ 35

In the eigenbasis of *Ĥ*_S_, eq 34 can be rewritten using the following matrix form:

$$S_{mn}^{\alpha }\left(t\right)=\left\langle n\right|{Ŝ}_{\alpha }\left(t\right)\left|m\right\rangle =S_{mn}^{\alpha }e^{i\omega_{mn}t}$$ 36

where ω_*mn*_ = ϵ_*m*_ – ϵ_*n*_, in which ϵ_*m*_ is the eigenvalue of *Ĥ*_S_ corresponding to the eigenstate |*m*⟩. Equation 34 is transformed in the Schrödinger picture as^228^ (the subscript S is omitted here):

$$\frac{d\rho_{ab}\left(t\right)}{dt}=-i\omega_{ab}\rho_{ab}\left(t\right)-\underset{\alpha ,\beta }{\sum }\underset{c,d}{\sum }\int_{0}^{\infty }dt^{\prime }\;\{g_{\alpha \beta }\left(t^{\prime }\right)[\delta_{b,d}\underset{n}{\sum }S_{an}^{\alpha }S_{cn}^{\beta *}e^{i\omega_{cn}t\prime }-S_{ca}^{\beta *}S_{db}^{\alpha }e^{i\omega_{ca}t\prime }]+g_{\alpha \beta }^{*}\left(t^{\prime }\right)[\delta_{a,c}\underset{n}{\sum }S_{dn}^{\beta }S_{bn}^{\alpha *}e^{i\omega_{nd}t\prime }-S_{ca}^{\alpha *}S_{db}^{\beta }e^{i\omega_{bd}t\prime }]\}\rho_{cd}\left(t\right)$$ 37

The first term on the right-hand arises from the static Hamiltonian *Ĥ*_S_, while the integral accounts for the spin relaxation induced by the system–bath interactions. Equation 37 can be rewritten in terms of the spectral densities *J*_αβ_(ω_*mn*_):^228^

$$J_{\alpha \beta }\left(\omega_{mn}\right)=\int_{0}^{\infty }dt^{\prime }\;g_{\alpha \beta }\left(t^{\prime }\right)e^{i\omega_{mn}t\prime }$$ 38

which represents the strength of the system–bath coupling at frequency ω_*mn*_. Equation 37 thus assumes the form^185^

$$\frac{d\rho_{ab}}{dt}=-i\omega_{ab}\rho_{ab}+\underset{c,d}{\sum }R_{abcd}\rho_{cd}$$ 39

where *R*_*abcd*_ is given by

$$R_{abcd}=-\underset{\alpha ,\beta }{\sum }[\delta_{b,d}\underset{n}{\sum }S_{an}^{\alpha }S_{cn}^{\beta *}J_{\alpha \beta }\left(\omega_{cn}\right)-S_{ca}^{\beta *}S_{db}^{\alpha }J_{\alpha \beta }\left(\omega_{ca}\right)+\delta_{a,c}\underset{n}{\sum }S_{dn}^{\beta }S_{bn}^{\alpha *}J_{\alpha \beta }^{*}\left(\omega_{dn}\right)-S_{ca}^{\alpha *}S_{db}^{\beta }J_{\alpha \beta }^{*}\left(\omega_{db}\right)]$$ 40

Including the reaction operator and transforming eq 39 into Liouville space, one obtains the following equation of motion for the spin density operator:

dρ̂(t)dt=−iH^^Sρ̂(t)−K^^ρ̂(t)+R^^ρ̂(t)=L^^ρ̂(t) 41

where L^^ is the total Liouvillian superoperator.

BRW theory is used to investigate several kinds of spin relaxation phenomena within the remit of the above-mentioned assumptions. Furthermore, BRW can be used for multiscale modeling by directly constructing correlation functions from molecular dynamics simulations of proteins and subsequent quantum chemical calculations of spin interactions.^76,184^ However, BRW theory certainly has its flaws and cannot be used in any situation. For instance, when the decay time of the correlation functions is much longer than the dynamics of the RP, BRW is not suitable. Furthermore, the Redfield relaxation matrix R^^ grows rapidly with the number of spins considered in the system, only allowing the usage of BRW for rather small SCRP spin systems.

Additionally, BRW theory, as most of the stochastic approaches, is not suitable when investigating the effect of RF-MFs, treated as a relaxation mechanism, as RF radiation most commonly has a periodic form over the whole dynamics of the SCRP and, thus, has much longer correlation times than the dynamics of the SCRP. BRW theory may be, however, used as an additional relaxation theory to include nonperiodic relaxation processes in theories that consider periodic Hamiltonians and can be combined with other relaxation theories. Furthermore, BRW was used in several studies to incorporate spin relaxation effects (mostly through isotropic rotational diffusion) of SCRPs within organic dyads and molecular wires^237–240^ at high fields where the inclusion of many nuclear spins was not necessary.

#### 11.5.4 Nakajima–Zwanzig Theory

An alternative to the BRW theory that is less commonly used is the Nakajima–Zwanzig (NZ) theory.^241–244^ As demonstrated by Fay et al., the NZ theory suffers significantly less when longer correlation times of the external perturbation are present while preserving a similar master equation compared to BRW.^245^ Fay et al. demonstrated the NZ theory in the context of stochastic nuclear coordinate distribution within molecules of an SCRP^245^ with a generalized set of coordinates *X* describing the nuclear motion. Similar to the BRW theory, the NZ theory is based on the fluctuations of spin–spin interactions within the full Hamiltonian:^245^

$${Ĥ}_{\mathrm{tot}}\left(X\right)={Ĥ}_{0}+\mathrm{V̂}\left(X\right)=\left\langle Ĥ\right\rangle +\underset{j}{\sum }f_{j}\left(X\right){Â}_{j}$$ 42

where *Ĥ*_0_ is the ensemble-averaged static part of the spin system and *V̂*(*X*) is the fluctuation part of a stochastic variable *X*, which again can be decomposed into scalar-valued functions *f*_*j*_(*X*) and spin-system-specific unitary operators *Â*_*j*_.^245^ Again, correlation functions can be obtained through *f*_*j*_(*X*) as

$$g_{jk}\left(\tau \right)=\left\langle f_{j}{\left(0\right)}^{*}f_{k}\left(\tau \right)\right\rangle =\int dX\;f_{k}{\left(X\right)}^{*}e^{\mathrm{D̂}\tau }f_{j}\left(X\right)p_{0}\left(X\right)$$ 43

where *D̂* is the operator describing the stochastic evolution and *p*_0_(*X*) is the equilibrium density of the system dependent on *X*.^245^ The formal approach of the NZ theory can be achieved by projecting the density of the system from the total density operator:^245^

$$\frac{\partial }{\partial t}P\mathrm{\rho ̂}\left(t,X\right)=PLP\mathrm{\rho ̂}\left(t,X\right)+\int_{0}^{t}d\tau \;K\left(t-\tau \right)P\mathrm{\rho ̂}\left(\tau ,X\right)$$ 44

where L=−i[Ĥ(X),×]−{K̂,×}+D̂ (with × being a placeholder) is the full Liouvillian of the complete system and P is the projection operator of the spin system of interest. Additionally, it is assumed that Pρ̂(0,X)=ρ̂(0,X) . The kernel K(t) is formulated as^245^

$$K\left(t\right)=PLQe^{QLt}QLP$$ 45

where Q=1−P is the bath projection operator. The definition of P in the case of stochastic fluctuations is^245^

$$PÔ\left(X\right)=p_{0}\left(X\right)\int dX^{\prime }\;Ô\left(X^{\prime }\right)$$ 46

where *Ô*(*X*) is an arbitrary operator depending on the variable *X*. The perturbation Louvillian of the spin system can be defined as $L_{v}=-i\left[\mathrm{V̂}\left(X\right),\times \right]$ and a reference Liouvillian is then obtained through $L_{0}=L-L_{v}$ . The kernel can now be expanded to the second order in $L_{v}$ and the Markovian approximation^184,219^ (similar to BRW) is employed, leading to the perturbative master equation for *P̂*ρ̂(*t*, *X*):

$$\frac{\delta }{\delta t}P\mathrm{\rho ̂}\left(t,X\right)=\left(L_{0}+K^{\left(2\right)}\right)P\mathrm{\rho ̂}\left(t,X\right)$$ 47

with

$$K^{\left(2\right)}=\int_{0}^{\infty }d\tau \;PL_{V}e^{L_{0}\tau }L_{V}P$$ 48

using the assumptions that $L_{0}P=PL_{0}$ and $PL_{V}P=0$ . After integrating out the stochastic variable *X*,^219^ the perturbative NZ master equation for an ensemble-averaged density operator is obtained:^245^

ddt⟨ρ̂(t)⟩=−i[Ĥ0,⟨ρ̂(t)⟩]+{K̂0,⟨ρ̂(t)⟩}+R^^NZ⟨ρ̂(t)⟩ 49

where the NZ relaxation superoperator is formulated as^245^

R^^NZ=∫0∞dτ∫dXLVeL0τLVp0(X)=−∑j,k∫0∞dτgjk(τ)Aj†e⟨L⟩τAk 50

with the superoperators $A_{j}^{†}=\left[{Â}_{j}^{†},\times \right]$ and $A_{k}=\left[{Â}_{k},\times \right]$ , and $\left\langle L\right\rangle =L_{0}-\mathrm{D̂}$ .

Fay et al. demonstrated that the NZ formalism is a more stable approach for the description of stochastic relaxation processes compared to the BRW theory. However, in the extreme narrowing limit, where correlation times are short, NZ and BRW are identical.^245^ Furthermore, NZ theory suffers from similar computational problems as BRW with a rapidly increasing dimensionality of the relaxation matrix depending on the number of included spins. Additionally, effects through RF radiation treated as relaxation cannot be considered within NZ theory, however, it is an ideal theory to be incorporated to consider stochastic fluctuations into other theories that will be explored in this review. Although the NZ formalism has been less commonly applied to SCRP systems, its advantages over the BRW approach are becoming increasingly evident in experimental contexts. For example, Roger et al. recently employed NZ theory to successfully model an organic donor–acceptor dyad connected via a triptycene bridge.^246^ In this system, slow conformational motions led to long correlation times under which conditions the BRW formalism is no longer valid, whereas the NZ approach remains applicable.

#### 11.5.5 Periodic Fluctuations

Numerous phenomena in physics exhibit periodicity in distinct physical quantities, such as time or space. This inherent periodic characteristic is frequently utilized in quantum mechanics as a tool for simplification, enabling the formulation of master equations. For example, the use of periodic characteristics is vital for the theoretical examination of complex systems, with solids serving as a prime example, as elucidated by Bloch.^247^ Similarly, EM waves and, consequently, RF-MFs demonstrate analogous periodicity while propagating through a system of interest, with the oscillation time period of the waves being the focal point of interest.

Motivated by the time periodicity inherent to EM waves, various theories have been developed^74,222,248–252^ in the context of spin dynamics. These theoretical frameworks efficiently transform the time-dependent Hamiltonian into their time-independent counterparts, making the equation of motion solution more approachable. In the subsequent sections, we shall delve into three notable approaches that are particularly relevant to the interaction between RF-MFs and SCRPs.

#### 11.5.6 Rotating Frame Approximation

The rotating frame approximation (RFA) can only be employed when the periodic time-dependency exhibits a rotational characteristic such as circularly polarized (CP) oscillating magnetic fields^183,212,222^ (linear polarized light can be also decomposed into two circularly parts and the RFA can be employed under certain assumptions). For CP oscillating magnetic fields, a transformation of the reference frame of the system can be exploited, which formally removes the time-dependency of the Hamiltonian. For the reference frame transformation, the vector *n⃗* will be used in the following as the rotation axis of the CP oscillating field. It is furthermore assumed that the total time-independent part of the spin Hamiltonian has rotational symmetry around *n⃗*.

The transformation of a time-dependent Hamiltonian into a time-independent Hamiltonian is equal to a coordinate transformation of the system. Here, the unitary operator for the transformation is defined as (ℏ = 1):

$$Û=e^{-i\omega Ĵ\cdot \mathrm{n⃗}t}$$ 51

where ω is the angular frequency of the rotation and *Ĵ* is the total spin operator for the involved electrons and nuclei. With this transformation, the LvN equation can be rewritten as

$$\frac{d}{dt}\left(Û\mathrm{\rho ̂}{Û}^{†}\right)=-i\left[ÛĤ{Û}^{†},Û\mathrm{\rho ̂}{Û}^{†}\right]+\left(\frac{dÛ}{dt}\right)\mathrm{\rho ̂}{Û}^{†}+Û\mathrm{\rho ̂}\left(\frac{d{Û}^{†}}{dt}\right)$$ 52

Here, two new terms appear containing the expression^253^

$$\frac{dÛ}{dt}=-i\omega Ĵ\cdot \mathrm{n⃗}Û$$ 53

The transformed LvN equation can then be reformulated as

$$\frac{d\mathrm{\rho ̃}}{dt}=-i\left[\mathrm{H̃},\mathrm{\rho ̃}\right]$$ 54

where the transformed Hamiltonian *H̃* = *ÛĤÛ* ^†^ – *ωĴ*·*n⃗* now has an additional term describing a fictitious interaction that accounts for the motion of the reference frame, similar to the centrifugal force in classical mechanics. The RFA is advantageous when investigating monochromatic circularly polarized light but becomes unsuitable for linearly polarized broadband radiation, which is often the case for anthropogenic RF fields, or when anisotropic magnetic interactions are present where the rotation axis is not parallel to the magnetic field axis.

#### 11.5.7 γ-COMPUTE

γ-COMPUTE (Computation Over one Modulation Period Using Time Evolution) was originally proposed in terms of solid-state NMR and the so-called magic angle spinning of solid-state samples.^254–256^ The γ in γ-COMPUTE has been introduced in the solid-state context as one of the Euler angles for the rotation of the sample in a solid-state NMR experiment. However, similar to the periodic spinning that the spin system experiences, the RF-MF can also be thought of as the periodic rotation experienced by an SCRP. Thus, the γ-COMPUTE formalism can be used for the consideration of RF radiation effects within a spin system by exploiting the periodic nature of the RF Hamiltonian. To derive the equation of the γ-COMPUTE algorithm, one starts with the time-dependent Hamiltonian of a spin system including, i.e., RF-MFs such as

$$Ĥ\left(t,\gamma \right)=\omega_{1}{Ŝ}_{Nj}\;\sin\left(\omega_{\mathrm{RF}}t+\gamma \right)$$ 55

where *Ŝ*_*Nj*_ is a component *j* of the spin operator of the spin *N*, ω_RF_ is the angular frequency of the considered RF field, $\omega_{1}=\frac{\gamma_{e}}{2\pi }B_{1}$ and γ is the phase of the radiation field at time *t* = 0, denoted as *t*_0_. The time evolution is performed with a time propagator *U*(*t*; *t*_0_, γ):^256,257^

$$U\left(t;t_{0},\gamma \right)=\mathrm{T̂}e^{-i\int_{t_{0}}^{t}Ĥ\left(t\prime ,\gamma \right)dt\prime }$$ 56

RPs are usually formed by continuous illumination in the presence of a continuous RF field which in turn leads to the creation of a specific SCRP at any point of time during a whole cycle of the RF field. Thus, the observable of interest (e.g., *P̂*_*S*_) must be averaged over a uniform distribution of the phase γ in the interval of [0, 2π). Here, one can exploit the symmetry properties of the RF field to simplify the propagation of the SCRP system. The RF fields are periodic in time:^257^

$$Ĥ\left(t,\gamma \right)=Ĥ\left(t+\frac{2m\pi }{\omega_{\mathrm{RF}}},\gamma \right),\forall m\in Z$$ 57

and the phase γ can be considered as a time shift of the sinusoidal behavior of the RF field leading to

$$Ĥ\left(t,\gamma \right)=Ĥ\left(t+\frac{\gamma }{\omega_{\mathrm{RF}}},0\right)$$ 58

The symmetries in eqs 57 and 58 mean that the time evolution of the spin system may be divided into contributions from each whole RF period *T*:

$$T=\frac{2\pi }{\omega_{\mathrm{RF}}}$$ 59

plus a residual contribution from times after the last complete RF period. The propagator *U*(*T*;0, γ) is the central operator for the description of the spin dynamics under the influence of an RF field,^257^ which can be solved using the symmetry relations in eq 57 and ([Disp-formula eq58]). The population of a specific spin state, e.g., the singlet state *P̂* ^*S*^, also contains contributions from each whole RF period with additional contributions arising because of time evolution during the final partially completed RF period.

With the properties above in mind, it is possible to discretize the Hamiltonian. Here, the continuously varying Hamiltonian is approximated with one that is constant within time intervals. Dividing one period *T* of the RF field in eq 59 into *n* time intervals of duration τ,^256^

$$\tau =\frac{T}{n}$$ 60

gives the possibility to discretize the problem using integer indices *j* and *p*:

$$j=\frac{t}{\tau }$$ 61

$$p=\frac{\gamma }{2\pi /n}$$ 62

The integer value *j* is used for the time discretization, while the value *p* is used for the phase γ. Using the integer indices, the propagator *U* for a time period 0 → *j*τ with phase γ = 2π*p*/*n* $\gamma =p\frac{2\pi }{n}$ can be reformulated as

$$A\left(j,p\right)=U\left(j\tau ;0,\frac{2\pi p}{n}\right)$$ 63

Once *n* is sufficiently large, the periodic Hamiltonian *Ĥ*(*t*, γ) varies negligibly within each time step τ, and hence, the integral in eq 56 may be approximated by the followingproduct:

$$A\left(j,p\right)\approx e^{-i\tau Ĥ\left(\left[j-\frac{1}{2}\right]\tau ,\frac{2\pi p}{n}\right)}\cdot \cdot \cdot e^{-i\tau Ĥ\left(\frac{\tau }{2},\frac{2\pi p}{n}\right)}$$ 64

except for time *t* = 0 where A(0,p)=I .^257^ By taking the midpoint value of each time step of *Ĥ* in eq 64, the error arising from the approximation of discretization can be reduced.^257^ Note that the order of the exponential terms in eq 64 is due to the acting of an operator on a state vector. Furthermore, by considering the symmetry relations of eqs 57 and 58, the new operator *A*(*j*, *p*) can be formulated in terms of *A*(*j*, 0) = *A*(*j*) where the phase of the RF field is zero:^257^

$$A\left(j,p\right)=A\left(j+p\right)A{\left(p\right)}^{†}=A\left(j+p\;\mathrm{mod}n\right)A{\left(n\right)}^{m}A{\left(p\;\mathrm{mod}n\right)}^{†}$$ 65

with

$$m=\left\lceil \frac{j+p}{n}\right\rceil -\left\lceil \frac{p}{n}\right\rceil$$ 66

where ⌈*x*⌉ is the largest integer less than or equal to *x* and mod is the modulo function.^257^ Thus, any *A*(*j*, *p*) may be expressed in terms of *n* propagators *A*(1···*n*). The γ-COMPUTE algorithm calculates all *n* propagators sequentially at the start and evaluates each matrix exponential in eq 64 only once. Furthermore, several other steps such as averaging the Hamiltonian and the RF phase or evaluating the transition frequencies ω_*rs*_^′^, where *r* and *s* are the eigenstates of the SCRP, have to be made to derive the final expression for the expectation value.^257^

The master equation for a population of a state of interest Θ under the influence of a periodic RF-MF for the γ-COMPUTE algorithm is usually solved in the frequency domain rather than the time domain:^257^

$$\left\langle {\mathrm{P̂}}_{\Theta }\right\rangle =\overset{4M}{\underset{r,s=1}{\sum }}\overset{n/2-1}{\underset{k=-n/2}{\sum }}{\left|G_{rs}\left(k\right)\right|}^{2}\delta \left(\omega +\omega_{rs}^{\prime }+k\omega_{\mathrm{RF}}\right)$$ 67

where *M* is the dimension of the nuclear spin space, |*G*_*rs*_(*k*)|^2^ are cross-correlation functions derived through the discrete Fourier transform (DFT) of the periodic function *g*_*rs*_(*x*), which combines the effects of the modulation frequency ω_*rs*_, the projection *P̂*_*rs*_^Θ*T*^(*x*) (transformed into the eigenbasis of the averaged zero-phase RF Hamiltonian), and the periodicity *n*.^257^ Specifically, *g*_*rs*_(*x*) is defined as

$$g_{rs}\left(x\right)=e^{-i\omega_{rs}\left(x\mathrm{mod}n\right)\tau }{\mathrm{P̂}}_{rs}^{\Theta T}\left(x\right)$$ 68

and

$$G_{rs}\left(k\right)={\mathrm{DFT}}_{k}\left(\overset{n-1}{\underset{p=0}{\sum }}g_{rs}^{*}\left(p\right)g_{rs}\left(j+p\right)\right)$$ 69

where *x* represents a discrete time index variable that increments as the system evolves. The squared magnitude |*G*_*rs*_(*k*)|^2^ represents the contribution of the *k*th harmonic of the RF field modulation to the quantum yield of the spin-selective reaction. Furthermore, in eq 67 ω_RF_ is the frequency of the applied RF-MF, and ω is the frequency corresponding to the time *t* through the Fourier transform. The quantum yield of a spin-selective reaction can then be evaluated by using eq 67:

$$\Phi_{\Theta }=\int_{0}^{\infty }\left[\frac{\sqrt{2\pi }}{Mn^{2}}\overset{4M}{\underset{r,s=1}{\sum }}\overset{n/2-1}{\underset{c=-n/2}{\sum }}{\left|G_{rs}\left(c\right)\right|}^{2}\delta \left(\omega +\omega_{rs}^{\prime }+c\omega_{\mathrm{RF}}\right)\right]F{\left(\omega \right)}^{*}d\omega =\frac{\sqrt{2\pi }}{Mn^{2}}\overset{4M}{\underset{r,s=1}{\sum }}\overset{n/2-1}{\underset{k=-n/2}{\sum }}{\left|G_{rs}\left(k\right)\right|}^{2}F\left(\omega_{rs}^{\prime }+k\omega_{\mathrm{RF}}\right)$$ 70

where *F*(ω) is the Fourier transform of a re-encounter probability distribution *f*(*t*).^257^ An excellent in-depth derivation of the γ-COMPUTE algorithm to evaluate the quantum yield of a spin-selective reaction for a correlated SCRP can be found in the work of C. Rodgers.^257^ Although considering periodic oscillating interactions through the construction of multiple propagators for a full modulation period is an elegant procedure, the γ-COMPUTE algorithm rapidly becomes unusable due to the large number of matrix exponentials that are required to construct the propagator when the RF field contains many Fourier components, especially when considering several nuclei spins.^250^ Nevertheless, the γ-COMPUTE algorithm was successfully employed for verifying a diagnostic test for the RPM within the radical ion pair chrysene and dicyanobenzene using radiofrequency radiation with an amplitude of 300 μT in the range of 5–50 MHz.^252^

#### 11.5.8 Floquet Theory

Floquet theory is the time analog to the spatial Bloch theory and provides a powerful approach for analyzing quantum systems with time-periodic Hamiltonians; it was introduced in the context of SCRP dynamics by Hiscock et al.^250^ The theory is particularly useful for systems described by a Hamiltonian *Ĥ*(*t*) that oscillates with an angular frequency ω, as one expects in the case of RF-MFs. In the Floquet framework, the Hamiltonian is expanded as a Fourier series:

$${Ĥ}_{F}\left(t\right)=\overset{\infty }{\underset{n=-\infty }{\sum }}{Ĥ}^{\left(n\right)}e^{in\omega t}$$ 71

where *Ĥ* ^(*n*)^ are the Fourier components of the Hamiltonian.^250^ Floquet theory introduces an extended space known as the Floquet space, described in great detail by Shirley,^258^ which is a product of Hilbert space basis states |α⟩ and Fourier space basis states |*m*⟩. The Floquet space is often introduced to exchange the time-dependency problem of a Hamiltonian for an infinite time-independent dimension problem which will be then truncated by numerical approaches.^259^ A basis state in the new Floquet space is denoted as |α, *m*⟩.^250^ The theory allows expressing the matrix elements of the time-evolution operator *U*_βα_(*t*; *t*_0_) within the range [*t*; *t*_0_] as follows:^250^

$$U_{\beta \alpha }\left(t;t_{0}\right)=\underset{n}{\sum }\left\langle \beta ,n\right|e^{-i{Ĥ}_{F}\left(t-t_{0}\right)}\left|\alpha ,0\right\rangle e^{in\omega t}$$ 72

where *t*_0_ is the initial time of the system of interest, and *Ĥ*_*F*_ is the Floquet Hamiltonian.^250^ The eigenvalues *ε*_*l*_ and eigenstates |*ε*_*l*_⟩ of the Floquet Hamiltonian *Ĥ*_F_ have specific periodic properties:^250^

$$\varepsilon_{\gamma ,l+p}=\varepsilon_{\gamma ,l}+p\omega$$ 73

$$\left\langle \alpha ,n+p\right|\varepsilon_{\gamma ,l+p}\rangle =\left\langle \alpha ,n\right|\varepsilon_{\gamma ,l}\rangle$$ 74

where *p* and *l* are integers representing Fourier modes.^250^ These relations are required to ensure the unitarity of the propagator *U*_*βα*_ and are used to reformulate *U*_βα_(*t*; *t*_0_) as

$$U_{\beta \alpha }\left(t;t_{0}\right)=\underset{n,\gamma ,l}{\sum }\left\langle \beta ,n\right|\varepsilon_{\gamma ,l}\rangle e^{-i\varepsilon_{\gamma ,l}\left(t-t_{0}\right)}\left\langle \varepsilon_{\gamma ,l}\right|\alpha ,0\rangle e^{in\omega t}=\underset{n,\gamma ,l}{\sum }\left\langle \beta ,0\right|\varepsilon_{\gamma ,l-n}\rangle e^{-i\varepsilon_{\gamma ,l-n}\left(t-t_{0}\right)}\left\langle \varepsilon_{\gamma ,l-n}\right|\alpha ,\left(-n\right)\rangle e^{in\omega t_{0}}=\underset{n,\gamma ,m}{\sum }\left\langle \beta ,0\right|\varepsilon_{\gamma ,m}\rangle e^{-i\varepsilon_{\gamma ,m}\left(t-t_{0}\right)}\left\langle \varepsilon_{\gamma ,m}\right|\alpha ,\left(-n\right)\rangle e^{i\left(-n\right)\omega t_{0}}=\underset{n}{\sum }\left\langle \beta ,0\right|e^{-i{Ĥ}_{F}\left(t-t_{0}\right)}\left|\alpha ,n\right\rangle e^{in\omega t_{0}}$$ 75

The expectation value of an arbitrary observable *A* in terms of the density matrix formalism at a time instance *t* can be calculated using initial conditions specified at a time instance *t*_0_ through Floquet space, which would usually be in the density operator formalism:

$$\left\langle A\left(t;t_{0}\right)\right\rangle =\mathrm{Tr}\left[ÂÛ\left(t;t_{0}\right)\mathrm{\rho ̂}\left(t_{0}\right){Û}^{†}\left(t;t_{0}\right)\right]$$ 76

Equation 76 is evaluated in the Floquet space in terms of the Floquet space detection operator:

$$\left\langle A\left(t;t_{0}\right)\right\rangle ={\mathrm{Tr}}_{F}\left[{Â}_{F}e^{-i{Ĥ}_{F}\left(t-t_{0}\right)}{\mathrm{\rho ̂}}_{F}\left(t_{0}\right)e^{i{Ĥ}_{F}\left(t-t_{0}\right)}\right]$$ 77

where ρ̂_F_(*t*_0_) is the Floquet density matrix, defined as

$${\mathrm{\rho ̂}}_{F}\left(t_{0}\right)=\underset{\alpha ,\beta }{\sum }\underset{m,n}{\sum }\left|\alpha ,m\right\rangle e^{-im\omega t_{0}}\rho_{\alpha \beta }\left(t_{0}\right)e^{in\omega t_{0}}\left\langle \beta ,n\right|$$ 78

while the Floquet space detection operator is defined as^250^

$${Â}_{F}=\underset{\alpha ,\beta }{\sum }\left|\alpha ,0\right\rangle A_{\alpha \beta }\left\langle \beta ,0\right|$$ 79

Furthermore, it is convenient to specify the initial condition at a time instance *t*_0_ = 0, in which case the phase factors usually found in the initial density operator ρ̂_F_ disappear and eqs 77 and 78 simplify to^250^

$$\left\langle A\left(t\right)\right\rangle ={\mathrm{Tr}}_{F}\left[{Â}_{F}e^{-i{Ĥ}_{F}t}{\mathrm{\rho ̂}}_{F}\left(0\right)e^{i{Ĥ}_{F}t}\right]$$ 80

where

$${\mathrm{\rho ̂}}_{F}\left(0\right)=\underset{\alpha ,\beta }{\sum }\underset{m,n}{\sum }\left|\alpha ,m\right\rangle {\mathrm{\rho ̂}}_{\alpha \beta }\left(0\right)\left\langle \beta ,n\right|$$ 81

By finding or approximating the eigenvalue spectrum of *Ĥ*_F_, one can evaluate the expectation value of ⟨*A*(*t*)⟩ by inserting the resolution of the identity in the Floquet space as

$$\left\langle A\left(t\right)\right\rangle =\underset{\alpha ,\beta }{\sum }\underset{m,n}{\sum }\left\langle \varepsilon_{\alpha ,m}\right|{Â}_{F}\left|\varepsilon_{\beta ,n}\right\rangle \left\langle \varepsilon_{\beta ,n}\right|{\mathrm{\rho ̂}}_{F}\left(0\right)\left|\varepsilon_{\alpha ,m}\right\rangle e^{i\left(\varepsilon_{\alpha ,m}-\varepsilon_{\beta ,n}t\right)}$$ 82

As stated before, while the time dependency can be handled rigorously, the solution of the Floquet space problem is still an infinite-dimensional problem. Truncation of the problem has to be made numerically, i.e., solution approaches could be found by employing degenerate perturbation theory.^250^ Here, qualitative results for weak MF ≥ 25 nT were calculated first in simplified models. However, the memory required for the calculations increases rapidly, limiting the Floquet approach by Hiscock et al. to smaller spin systems if inter-radical interactions have to be taken into account. For nonperiodic spin relaxation processes, such as protein motion in a biological system, an additional modified relaxation term has to be included in the Floquet equation (written in superoperator notation):^260^

ddtρ̂F=−iH^^Fρ̂F(t)+Γ^^Fρ̂F(t) 83

where Γ^^F is the Floquet relaxation operator, in which stochastic relaxation theories such as BRW or NZ theory might be incorporated. The Floquet formalism in the density operator framework is a suitable theory to describe RF-MF effects on SCRPs. However, similar problems, such as the limited amount of nuclear spins that could be considered arise in all density operator formalisms. A wavefunction-based formalism based on Floquet theory that furthermore truncates the size problem by, e.g., stochastically evaluating the trace^261^ has not been reported yet for SCRP dynamics. The here derived Floquet formalism was used by Hiscock et al. to study potential magnetic field effects through weak monochromatic and broadband RF-MFs (1.4–80 MHz) within the cryptochrome protein of *Arabidopsis thaliana* cryptochrome.^75^ However, to our knowledge no direct comparison between an experimental result and Floquet theory was made for SCRPs so far.

#### 11.5.9 Monte Carlo Trace-Sampled Stochastic Schröd­inger Equation

Most of the previously discussed formalisms had one common motivation: to avoid the explicit description of fluctuations due to their complexity and numerical infeasibility. Furthermore, these frameworks were set up using the density matrix formalism. In many cases, they extend to the Liouville space, which indeed simplifies many aspects. This includes the ensemble description of the spin system, the straightforward relationship between the quantum system of interest and its external perturbation, and the suppression of nontrivial commutator terms.

However, the significant disadvantage of the density matrix formalism is its rapidly increasing resource demand, which limits all spin dynamics simulations to only a few spins. This is a fundamental drawback when investigating biological systems, which commonly consist of several atoms exhibiting spin. Previous works demonstrated the importance of including many nuclear spins within the spin system to describe the system as realistically as possible and that drastic changes in the calculated observables appear as the number of nuclear spins increases.^75–77^ There are semiclassical approaches that can be employed to incorporate many nuclear spins in the density matrix formalism, such as the Schulten-Wolynes theory^262^ or the improved semiclassical Manolopoulos-Hore theory.^263^ However, these theories cannot be straightforwardly used when the nuclear spins of interest, or more precisely, the hyperfine interactions between the electrons and nuclei, are time-dependent and these time-dependencies are of major importance for a possible RPM.

The wavefunction formalism for SCRP spin dynamics is only rarely employed even though the phase space (Hilbert space) is significantly smaller and thus in principle more spins can be considered.^71,199,264^ To evaluate ensemble dynamics similar to the density matrix formalism, several state vectors have to be propagated. The number of required state vectors increases rapidly with the included number of spins, similar to the density matrix formalism (state vectors are a sum over the distribution of nuclear spin configurations). Additionally, the time propagation of the state vectors has to be done explicitly for several time steps, making the wavefunction formalism similar to or more expensive than many approaches within the density matrix formalism. Furthermore, the direct coupling to an external bath in terms of open-quantum systems is nontrivial in the framework of the wavefunction formalism, eventually leading to the popularity of the density matrix formalism.

Fay et al.^199^ recently presented an elegant formalism to avoid propagating all state vectors of the spin system explicitly by stochastically evaluating the configuration space of nuclear spin states using Monte Carlo (MC) sampling in a so-called trace-sampled approach.^261^ Using the trace-sampled approach, it was possible to investigate systems with up to 20 spins.^199,265^ Furthermore, it was possible to treat time-dependent fluctuations of any interaction explicitly or stochastically, which were further exploited in recent studies.^198,266^ Thus, stochastic trace sampling is a promising candidate for the investigation of SCRP dynamics within complex biological environments.

To evaluate the equation of motion, a reformulation of the quantum-mechanical trace in eq 4 expanded in the basis of ref 267 is employed:

$$\left\{B\right\}=\left\{\left|\Theta \right\rangle ⊗\left|M_{1}\right\rangle ⊗\left|M_{2}\right\rangle \right\}$$ 84

Here |Θ⟩ describes the SCRP spin states and |**M**_*i*_⟩ describes the nuclear spin states of radical *i*, given by

$$\left|M_{i}\right\rangle =\left|M_{i1}\right\rangle ⊗\left|M_{i2}\right\rangle ⊗\cdot \cdot \cdot ⊗\left|M_{iN_{i}}\right\rangle$$ 85

where *M*_*ik*_ is the magnetic quantum number of the *k*th nuclear spin in radical *i*. An expansion of the trace in eq 4 with eq 84 by summing over all possible nuclear spin states leads to^265^

$$P_{\Theta }\left(t\right)=\mathrm{Tr}\left[I{\mathrm{P̂}}_{\Theta }\mathrm{\rho ̂}\left(t\right)I\right]=\mathrm{Tr}\left[\underset{\Theta ,\Theta^{\prime }}{\sum }\underset{M_{1},M_{1}^{\prime }}{\sum }\underset{M_{2},M_{2}^{\prime }}{\sum }\left|\Theta ,M_{1},M_{2}\right\rangle \langle \Theta ,M_{1},M_{2}|{\mathrm{P̂}}_{\Theta }\mathrm{\rho ̂}\left(t\right)\left|\Theta^{\prime },M_{1}^{\prime },M_{2}^{\prime }\right\rangle \langle \Theta^{\prime },M_{1}^{\prime },M_{2}^{\prime }|\right]=\underset{\Theta }{\sum }\underset{M_{1}}{\sum }\underset{M_{2}}{\sum }\left\langle \Theta ,M_{1},M_{2}\right|{\mathrm{P̂}}_{\Theta }\mathrm{\rho ̂}\left(t\right)\left|\Theta ,M_{1},M_{2}\right\rangle =\underset{\Theta }{\sum }\underset{M_{1}}{\sum }\underset{M_{2}}{\sum }\left\langle \Theta ,M_{1},M_{2}\right|Û{\left(t,0\right)}^{†}{\mathrm{P̂}}_{\Theta }Û\left(t,0\right)\mathrm{\rho ̂}\left(0\right)\left|\Theta ,M_{1},M_{2}\right\rangle =\frac{1}{Z}\underset{M_{1}}{\sum }\underset{M_{2}}{\sum }\left\langle \Theta_{\mathrm{init}},M_{1},M_{2},t\right|{\mathrm{P̂}}_{\Theta }\left|\Theta_{\mathrm{init}},M_{1},M_{2},t\right\rangle$$ 86

The transition from the first to the second line in the above equation is accomplished by exploiting the definition of ρ̂(*t*), as outlined in eq 5, and employing the properties of the trace (allowing for cyclic permutation). Since ρ̂(0) = $\frac{1}{Z}\left|\Theta_{\mathrm{init}}\right\rangle \langle \Theta_{\mathrm{init}}|⊗I_{Z}$ (eq 7), the sum over Θ vanishes with only Θ_init_ state remaining. Lastly, the property |Θ_init_, **M**_1_, **M**_2_, *t*⟩ = *Û*(*t*, 0)|Θ_init_, **M**_1_, **M**_2_⟩ is employed. If an exact solution such as shown in eq 86 is required, the trace is explicitly evaluated by summing over all possible nuclear spin states.^267^

The states |Θ_init_, **M**_1_, **M**_2_, *t*⟩ are subject to the dynamics of the Schrödinger equation:^199^

$$\frac{d}{dt}\left|\Theta_{\mathrm{init}},M_{1},M_{2},t\right\rangle =\left(-iĤ\left(t\right)-\mathrm{K̂}\left(t\right)\right)\left|\Theta_{\mathrm{init}},M_{1},M_{2},t\right\rangle$$ 87

The formalism as presented here is efficient for small spin systems but becomes infeasible when dealing with thousands of possible nuclear spin states. The direct evaluation would require *Z* state vectors to be propagated to compute the trace in eq 4. However, as shown by Fay et al.,^199^ a good approximation can be employed by using the stochastic evaluation of the trace, as demonstrated by Weiße et al.,^261^ over all possible nuclear spin states. This is achieved by defining the resolution of the identity (RI) of normalized nuclear spin states $I_{Z}\left|\psi \left(\xi \right)\right\rangle$ , parametrized by ξ:^199^

$$I_{Z}=Z\int d\xi \;p\left(\xi \right)\left|\psi \left(\xi \right)\right\rangle \left\langle \psi \left(\xi \right)\right|$$ 88

where *p*(ξ) is the normalized probability density of ξ. By substituting the RI into eq 86, the trace of the observable of interest can be calculated as^199^

$$P_{\Theta }\left(t\right)=\int d\xi \;p\left(\xi \right)\left\langle \Theta_{\mathrm{init}},\psi \left(\xi ,t\right)\right|{\mathrm{P̂}}_{\Theta }\left|\Theta_{\mathrm{init}},\psi \left(\xi ,t\right)\right\rangle$$ 89

where |Θ_init_, ψ­(ξ, 0)⟩ = |Θ_init_⟩⊗|ψ­(ξ)⟩. The time propagation of |Θ_init_, ψ­(ξ, *t*)⟩ follows the Schrödinger equation similarly to eq 87.

Different choices exist for the evaluation of the nuclear sample states.^199,267^ However, the SU­(Z) coherent states denoted as |**Z**⟩, where **Z** is a vector of complex numbers *Z*_*n*_ = *X*_*n*_ + i*Y*_*n*_ and *X*_*n*_ and *Y*_*n*_ are sampled from the distribution $p\left(Z\right)=\delta \left(|Z|-1\right)/S_{2Z}$ (where $S_{2Z}$ is the surface area of a 2*Z*-dimensional hypersphere of unit radius), were demonstrated to be an efficient choice.^199,266^ The |**Z**⟩ state in a chosen basis is then given by

$$\left|Z\right\rangle =\overset{Z}{\underset{n=1}{\sum }}\left|n\right\rangle Z_{n}$$ 90

with the constraint ⟨**Z**|**Z**⟩ = 1. These states have self-averaging properties due to the invariance of this distribution under unitary transformations of the vector **Z** (**Z** → **UZ**).^199^

The main advantage of this formalism is that only a certain number *M* of MC sampled states are required to be propagated in solving the trace, compared to *Z* wave packets in the explicit form in eq 87. However, *Z* ≫ *M*, and *Z* must be large to be computationally efficient, which can be implicitly explained by the loss of significance of a single nuclear spin state configuration when thousands of other possible configurations exist. Thus, the trace sampled Schrödinger equation is only viable for larger spin systems which, however, are mostly the case in biological systems.

The explicit and stochastic methods rely on time evolution of state vectors by the Schrödinger equation. For a generic state |Ψ­(*t*)⟩, the Schrödinger equation reads as

$$\frac{d}{dt}\left|\Psi \left(t\right)\right\rangle =\left(-iĤ\left(t\right)-\mathrm{K̂}\left(t\right)\right)\left|\Psi \left(t\right)\right\rangle$$ 91

and its solution with *t*_0_ being the initial time instance is given as

$$\left|\Psi \left(t_{0}+dt\right)\right\rangle =Û\left(t_{0}+dt,t_{0}\right)\left|\Psi \left(t_{0}\right)\right\rangle$$ 92

The time propagator *Û* can be approximated without the time-ordering operator using the first-order Magnus expansion for small time differences *t*_1_ – *t*_0_ = d*t*:^268^

$$Û\left(t_{0}+dt,t_{0}\right)\approx \exp\left[\int_{t_{0}}^{t_{0}+dt}d\tau \;\left(-iĤ\left(\tau \right)-\mathrm{K̂}\left(\tau \right)\right)\right]=\exp\left[-i\mathrm{\Omega ̂}\left(t_{0}+dt,t_{0}\right)dt\right]$$ 93

where the generator Ω, used for evolving the spin state, is given by^199,265^

$$\Omega \left(t_{0}+dt,t_{0}\right)={Ĥ}_{0}-i\mathrm{K̂}+\frac{1}{dt}\underset{j}{\sum }{Â}_{j}\int_{t_{0}}^{t_{0}+dt}f_{j}\left(\tau \right)\;d\tau$$ 94

Here, the fluctuating interactions of the spin system are described as a decomposition of a time-dependent fluctuation *f*_*j*_(τ) and the unitary operators *Â* similar to eq 42. The fluctuating functions *f*_*j*_(τ) can be integrated in various ways depending on the specific problem of interest. The evolution can then be performed using different approaches, e.g., the Arnoldi algorithm^269^ or the short-iterative Lanczos methods.^270^

Thus, the fluctuations *f*_*j*_(τ), which induce spin relaxation in an SCRP, will be explicitly considered in the propagation procedure, allowing direct consideration of multiple relaxation mechanisms simultaneously without further approximations. Hence, it is possible to incorporate an effect of many nuclear spins while also including RF-MF effects, motion-induced modulation of hyperfine, dipolar or exchange interaction, and other dephasing mechanisms. Furthermore, direct time trajectories of specific interactions extracted from molecular dynamics and quantum chemistry simulations may be incorporated into the MC-trace-sampled Schrödinger equation method, making it viable for multiscale modeling.^265^

The disadvantage of the MC-trace-sampled Schrödinger equation method is the numerical evaluation of the time-propagation, which becomes increasingly challenging if the lifetime of the SCRP is extraordinarily long (>10 μs). However, recently developed approximations to truncate the integration^265^ are used to mitigate the numerical problem. While being a relatively novel method for the investigation of the RPM mechanism, the MC-trace-sampled theory is a potential framework to incorporate all the required aspects for the dynamics of an SCRP within a biological system and the interaction with RF-MFs.

#### 11.5.10 Time-Dependent Interaction Theories: Which One to Use?

The theoretical exploration of the RPM and the potential impacts of weak RF-MFs in biological systems presents a formidable challenge. This task necessitates a thorough understanding of various critical factors: the array of fundamental spin interactions, spin-selective reaction pathways and lifetimes, and spin relaxation processes in the form of complex time dependencies of spin interactions.

As delineated in this review, all interactions and spin-selective reaction pathways are instrumental in accurately depicting an SCRP and its dynamics within a realistic context. Commonly utilized simplistic ”toy model” systems often omit numerous features, leading to unreliable results that are rather far from any realistic system. For example, in magnetoreception, the discrete angular dependence of the singlet quantum yield in a cryptochrome SCRP is diminished when inter-radical interactions are taken into account.^207^ However, the requisite magnetic field dependency may reemerge under specific time-dependent fluctuations of these interactions,^198^ thereby highlighting the significance of constructing complex spin systems and comprehending the synergies among interactions, beyond the limited scope of toy models.

The accurate portrayal of complex time-dependencies continues to pose a significant challenge in theoretical studies of SCRP dynamics. Various methods have been adopted to address both periodic and nonperiodic time dependencies of spin interactions, as illustrated in Figure 9. Theories that describe nonperiodic time dependencies are often a robust choice to explore molecular motion-induced spin relaxation effects, but they are not viable in the context of including time-periodic effects such as RF-MFs. Furthermore, in a recent study, it was demonstrated that correlation times of protein motions can be large, leading to the breakdown of perturbative Markovian theories such as BRW.^77^ A direct comparison between BRW and NZ theory was provied by Fay et al., who demonstrated that both theories provide similar results if the correlation times are short. However, NZ tends to avoid the breakdown of BRW for longer correlation times for reaction yields.^245^ Besides that, the incorporation of nonperiodic time-dependencies when using a theory that exploits the periodicity of the Hamiltonian is a nontrivial challenge. Additionally, similar to electronic structure calculations, the expanding size of the Hilbert space for SCRPs in biological environments is a pressing issue that demands further research and development. The trace-sampled stochastic Schrödinger equation (SSE) emerges as a promising approach for large spin systems, accommodating both nonperiodic and periodic time dependencies. It was also demonstrated by Pazera et al. that the trace-sampling approach using an explicit time-trajectory of hyperfine interaction and the BRW theory produce similar results as long as correlation times are within the regime of BRW theory.^265^ However, the SSE method is not without drawbacks, such as extended integration times with increasing SCRP lifetimes, necessitating ongoing refinement. Further research, especially in terms of weak RF-MFs, using the trace-sampled SSE is necessary to allow for a more precise evaluation of RF-MF-affected SCRP dynamics. It is evident that, akin to the extensive parameter space in experimental work, theoretical studies require critical interpretation. Despite the challenges, the theoretical investigation of the RPM is imperative. In certain scenarios, general principles may be deduced even from smaller models, provided these principles align with the commonly accepted functioning of the RPM. Additionally, applying varied theories and methodologies to the same spin problem can lead to a unified conclusion, which should be corroborated by experimental research.

**Figure 9:**
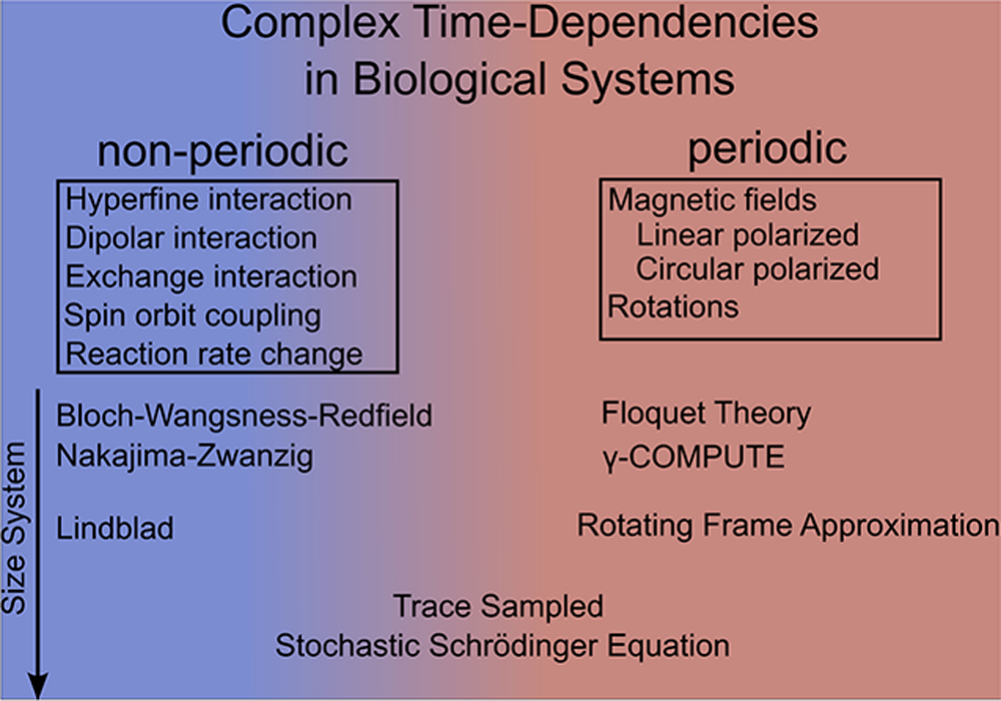
Comparison of different methods for the investigation of time-dependent interactions concerning the SCRP system size and property of the time-dependent interaction.

With these considerations in mind, an exploration into theoretical studies on the RPM and potential RF effects can be made. Afterward, a final discussion supported by the experimental and theoretical studies presented here will be employed, setting the state-of-the-art for the possible effects of weak RF-MFs on the biological systems under the paradigm of the RPM.

## 12 Theoretical Studies of RPM-Based RF-MF and ELF-MF Effects

The landscape of theoretical investigations focusing on the possible impact of weak RF-MFs on SCRP in biological systems remains sparse. Thus, here we also present studies on lower frequency domains, which also arise from anthropogenic sources. These are significant as they assist in providing a broader understanding of the impact of weak oscillating MFs on the RPM and allow the extraction of considerations that identically apply to future studies of possible RF-MF effects.

For monochromatic MFs at extremely low frequencies (ELF) around 50–60 Hz (which are generated by electrical appliances and power transmission lines and are associated with an increased risk of childhood leukemia^271^), a theoretical evaluation on a model SCRP was employed by Hore.^47^ Hore stated that at these low frequencies, the MFs will act on the short-lived SCRPs as a purely static field (one period of the MFs takes up to 20 ms). However, in a biological system, it can be assumed that SCRPs are continuously formed (with a lifetime much shorter than 20 ms) and that each formed SCRP experiences a different static MF, a feature that should also be considered in the case of RF-MFs. Thus, Hore used an ensemble average of the quantum yield Φ_T_(*B*_0_, *B*_1_) of an arbitrary triplet state selective reaction with randomly distributed MF strengths of a monochromatic ELF-MF with amplitude of *B*_1_ = 1 μT following the equation:^47^

$$B=B_{0}+B_{1}\;\cos\left(\alpha \right)$$ 95

where α ranges from 0 to π (randomly distributed) and *B*_0_ is the geomagnetic field experienced by the SCRP. The net effect of an ELF-MF on an ensemble of independently created SCRPs is then averaged over α and reads:^47^

$$\overset{®}{\Phi_{T}\left(B_{0},B_{1}\right)\;}=\frac{1}{\pi }\int_{0}^{\pi }\Phi_{T}\left(B\right)\;d\alpha$$ 96

Figure 10 illustrates the behavior of Φ_T_(*B*_0_, *B*_1_) with an applied ELF-MF in two different scenarios.^47^ As can be observed, as long as Φ_T_ responds linearly to the applied MF, essentially no difference can be found when considering an ELF-MF because through the oscillation of the MF both the maximal and the minimal amplitude (Figure 10A, orange) would average out.^47^ Only a difference is observed when the relation between Φ_T_(*B*_0_, *B*_1_) and the applied MF is nonlinear. In addition to that, Hore evaluated the MFE due to ELF-MF and compared it to the general fluctuations of the also applied geomagnetic field (GMF).^47^ Here, the MFE of the GMF exceeded the largest MFE of an ELF-MF by 2 orders of magnitude. Furthermore, Hore compared the fluctuations of the ELF-MFs to the fluctuations of Φ_T_(*B*_0_, *B*_1_) through thermal effects and estimated that for a temperature of *T* = 37 °C, a variation of ± 0.5 °C would have a similar effect to an ELF on the SCRPs quantum yield.^47^ Under the paradigm of the approximation made in the study, Hore proposed that the effects of 1 μT ELF-MFs in the presence of the GMF are small and similar to the same reactions resulting from other natural fluctuations in a biological system. However, he emphasized that the result is only valid under the assumption that the current theory of the RPM is complete.^47^ Furthermore, the SCRP model used did not involve many interactions (as certainly will be in biological systems) and only monochromatic ELF-MFs. Nevertheless, Hore was able to demonstrate how weak MFs with low frequency interact with a short-lived SCRP and emphasized important features such as the consideration of phase dependence for lower-frequency radiation.

**Figure 10:**
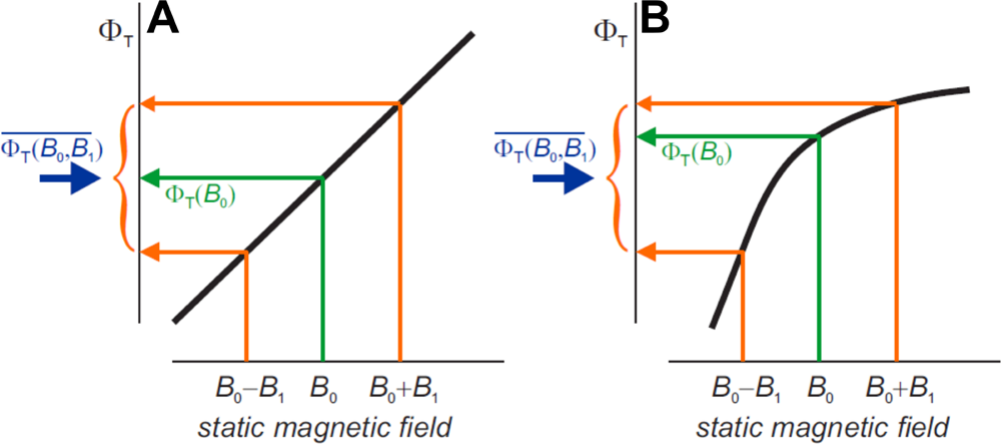
Illustration of the dependence of the reaction yield, Φ_T_, on the strength of magnetic field. The orange arrows show the yields for the maximal and minimal values of *B* in eq 95. The green arrow indicates the yields for only *B*_0_, while the blue arrows show the radical yields averaged over a full phase of the ELF field in eq 96. (A) When the quantum yield scales linearly with an increase in *B*, the combined effect of the ELF and the static field is equal to that of the static field alone. (B) When the quantum yield behaves nonlinearly, the effects of the static field + ELF and the static field alone differ. Reproduced from ref 47. CC BY 4.0.

Gauger et al. investigated the interaction of a toy model SCRP interacting with one nuclear spin, a static MF, and a weak (150 nT) RF-MF explicitly by including a time-dependent (ν_RF_ = 1.316 MHz) RF-MF.^73^ Additionally, spin relaxation through the Lindblad formalism was included in the calculations. They were motivated by the recently found disruption of the magnetic compass of migratory species through weak RF-MFs.^58,59,250^ In the studies by Gauger et al., it was revealed, similar to the study by Hore,^47^ that no significant effect of a weak RF-MF can be found when commonly used SCRP lifetimes are assumed. Only when using reaction rate constants of *k* = 10^4^ s^–1^, i.e., a lifetime of 100 μs, a significant change could be found. However, these extraordinarily long lifetimes (the important aspect is the coherence lifetime for the SCRP) are not expected for an SCRP within a biologically noisy environment.^73^ Furthermore, Gauger et al. investigated the effect of noisy environments and introduced spin relaxation on SCRP dynamics.^73^ Their calculations revealed that when the dephasing rate Γ ≥ *k*, the sensitivity of the SCRP to the angle dependence of an applied SMF drops drastically and that a decoherence time of 100 μs or more is required for an SCRP lifetime of 100 μs to produce anisotropy which is a mandatory property for the magnetic compass discussed in the concept of magnetoreception.^73,76,172^

Thus, the SCRP would be immune to an external weak RF-MF unless the coherence is preserved on a time scale of the order of 100 μs. The experimental results found in the context of magnetoreception in migratory species^73^ would therefore imply the unlikely fact that both amplitude and phase are protected within the avian compass.

Nielsen et al. demonstrated how to predict intracellular RF-MF effects on the RPM.^74^ In their study, a precise workflow was constructed to investigate the effect of RF-MFs on an SCRP embedded in a biological system such as a protein and listed defined criteria for the possible observation of RF-MF effects.^74^ Here, they stated four precise evaluations of a possible RF-MF effect would occur. Similar to Hore^47^ and Gauger et al.,^73^ the coherence lifetime of the SCRP is of crucial importance. If the characteristic time of an RF-MF action on the SCRP is much greater than its coherence lifetime, no RF-MF effect can be expected. The characteristic interaction time, τ_RF_ of the RF-MFs with the SCRP, can be determined once the strength of the RF-MF is known which can be estimated as^74^

$$\tau_{\mathrm{RF}}=\frac{2\pi \hbar }{g\mu_{B}B_{1}}$$ 97

Thus, τ_RF_ depends only on the field strength of the RF-MF, *B*_1_, but not on its oscillation frequency or polarization. In terms of weak RF-MFs, this means that the fields will not have sufficient time to interact with the SCRP before the SCRP decays, resulting in no RF-MF effect. Note that this rule only applies to monochromatic RF-MFs. Furthermore, inter-radical interactions should be evaluated.^74^ If inter-radical interactions are too large, no RF-MF effect will occur because the MFE itself is strongly suppressed. Nielsen et al. also emphasized that when the spin relaxation time is much shorter than the lifetime of the SCRP there will be no RF-MF effect.^74^ Lastly, they stated that the frequency of the RF-MFs has to be near a resonance frequency of the SCRP eigenvalue spectrum to induce a transition and trigger a response.

As an example, Nielsen et al. studied the SCRP [FAD^•–^–O_2_^•–^], which might be relevant for the increase of superoxide levels within cellular structures.^74^ They included eight nuclear spins from the FAD interacting with one of the SCRP’s electrons and ignored spin relaxation effects through, e.g., spin–orbit coupling. The lifetime of the SCRP was set to 1 μs (born in a singlet state), and an SMF of 50 μT was considered.^74^ Nielsen et al. used the rotating frame approximation to evaluate the effects of circularly polarized RF-MFs on the SCRP dynamics. Figure 11A,B illustrates the influence of the magnitude and frequency of an applied RF-MF on the singlet yield probability of the SCRP with and without an additional SMF of 50 μT, respectively. It can be observed that the presence of an additional weak SMF may have a significant effect on the quantum yields. The SMF changes the energy levels of the spin states in the SCRP, thereby changing the resonance frequencies of the spin system. At the Larmor frequency of 1.4 MHz, a significant change in singlet probability is observed (Figure 11C) due to the Zeeman interaction in the O_2_^•–^ radical, which has no other (stronger) interaction. In this context, the O_2_^•–^ radical can be considered as a free electron spin experiencing infinitely slow spin relaxation. However, significant changes in singlet probability considering an external MF are only observable at RF-MF magnitudes higher than 5 μT. However, without considering a static geomagnetic field, the effect of RF-MF at low intensities is striking. Over the whole frequency range in Figure 11B, a singlet probability change of more than 30% can be found. The significant change in singlet probability is explained through the eigenvalue spectrum of the spin system when no SMF is present. Here, the splitting of eigenstates through the external SMF is missing, and the resonance frequencies between the eigenstates are small.^74^ However, the conditions in Figure 11B are unlikely to occur in a common situation of a biologically relevant system due to the presence of the geomagnetic field. Nevertheless, so-called hypomagnetic field effects on SCRP systems are the focus of current research^69,74,117,118^ and can play an important role in the special situation of absence of the geomagnetic field.

**Figure 11:**
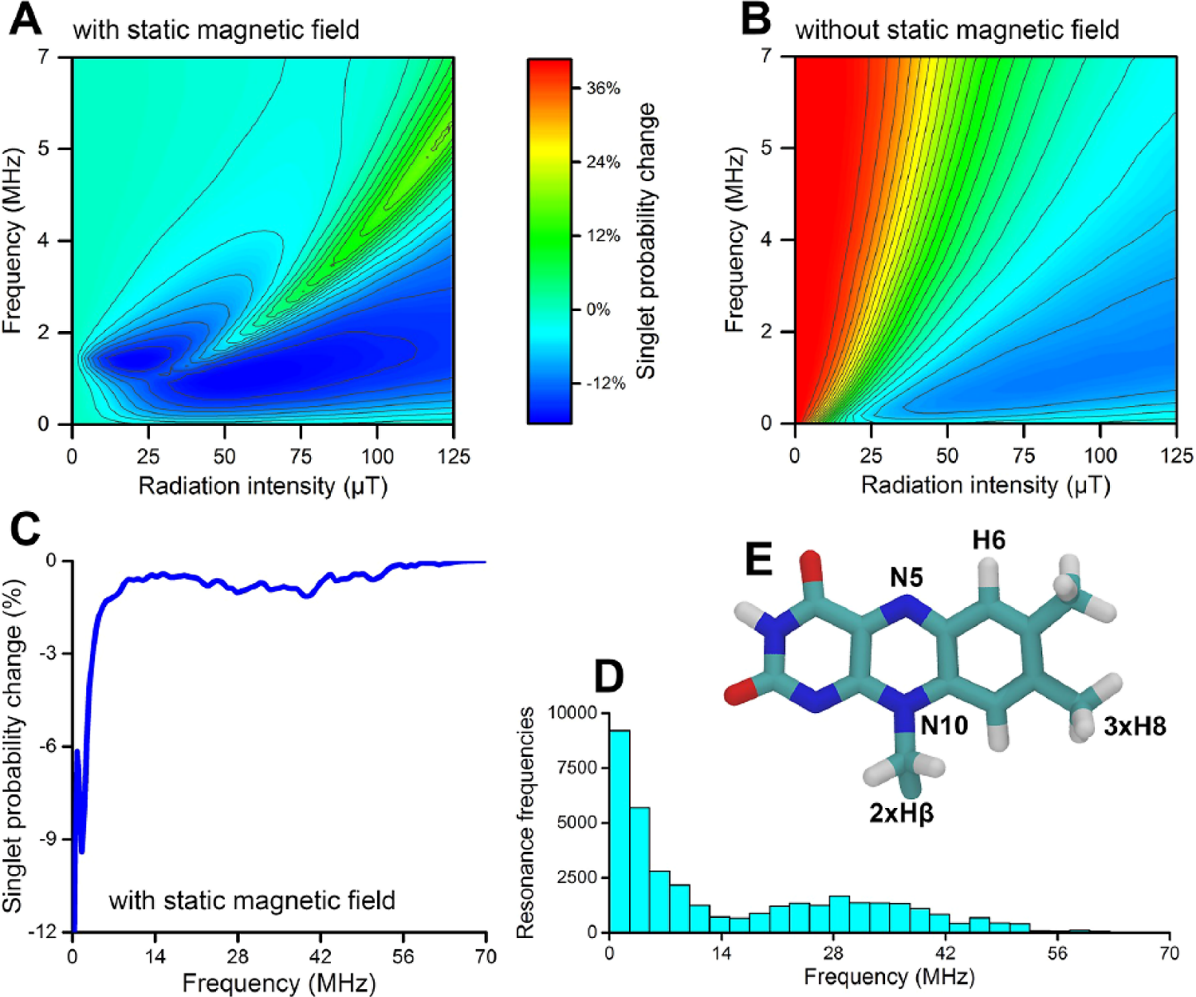
RF-MF effects in the [FAD^•–^–O_2_^•–^] SCRP. (A–C) Singlet product probability change (A) and (B) without an SMF (*B*_0_ = 50 μT). The reaction rate constants for singlet and triplet product formation were arbitrarily set so *k*_*S*_ = *k*_*T*_ = 1 μs^–1^. When the SMF is present, significant changes of singlet probability are only observable for frequencies below 7 MHz. D) Resonance frequencies of the SCRP system obtained from the eigenvalues of the spin Hamiltonian. E) Nuclear spins considered in the [FAD^•–^–O_2_^•–^] SCRP. Reproduced from ref 74. CC BY 4.0.

Another approach was used to qualitatively understand the possible influence of RF-MFs on an SCRP in three different studies using the action-histogram approach.^56,57,75^ In these studies, the static eigenvalue spectrum of the SCRPs Hamiltonian combined with possible transition probabilities was evaluated to allow for a qualitative expression of how RF-MFs might trigger transitions within the spin system. Here, the possible action or resonance effect *R*_effect_ of an external fluctuating MF was constructed using action histograms, where the heights of *n* bars, covering a frequency interval [*n*Δν, (*n* + 1)­Δν], are given by^75^

$$R_{\mathrm{effect}}\left(\nu ,\Delta n\right)=\underset{\nu_{ij}\in \left[n\Delta \nu ,\left(n+1\right)\Delta \nu \right]}{\sum }N{\left|\left\langle i\right|{Ĥ}_{⊥}\right|j\rangle |}^{2}\left|\left\langle i\right|{\mathrm{P̂}}_{S}\right|i\rangle -\left\langle j\right|{\mathrm{P̂}}_{S}\left|j\right\rangle |$$ 98

where *Ĥ*_⊥_ is the Zeeman Hamiltonian for a weak SMF perpendicular to the geomagnetic field, *P̂*_S_ is the singlet projection operator, |*i*⟩ and |*j*⟩ are the eigenstates of the time-independent spin Hamiltonian with an eigenvalue difference ν_*ij*_, and *N* is a normalization constant.^75^ Using eq 98, Leberecht et al. predicted the upper bound of frequencies that disturb the orientation of night-migratory songbirds, which were in good agreement with behavioral experiments using weak RF-MFs.^57^ However, the action-histogram approach only provides qualitative insight into possible transitions but cannot reveal details of the explicit dynamics of an SCRP under the influence of low-frequency RF-MFs.

Hiscock et al.^75,250^ used a more specific theoretical investigation of broadband RF-MF effects on the RPM using Floquet theory, focused on an SCRP within cryptochrome in the context of magnetoreception.^75^ As emphasized earlier, the consideration of nearby interacting nuclear spins and inter-radical interactions becomes extraordinarily important when investigating an SCRP in a protein. The more interactions are considered, the more eigenstates of the system appear, and RF-MFs at an arbitrary frequency below the cutoff frequency are more likely to induce a transition from one eigenstate to the other. Motivated by this, Hiscock et al. studied different SCRPs under the influence of RF-MF broadband exposure with an amplitude of 5.0 μT for monochromatic frequencies, while for broadband $M\times 50\times \frac{1}{\sqrt{2}}$ nT RF-MFs were used, where *M* is the number of frequency components within a frequency comb.^75^ In total, Hiscock et al. investigated four different SCRP systems with increasing complexity under the influence of broadband RF-MFs.^75^ In their most complex calculations of a FAD–tryptophan (FAD–Trp) SCRP found in cryptochrome (see Figure 12), they concluded that the overall quantum yield Φ of the SCRP is altered by the influence of broadband RF-MFs. However, there is no complete loss in sensitivity to the direction of the SMF.^75^ Furthermore, the authors used a coherent lifetime of the SCRP of 1 ms, which they qualified as implausible in the case of cryptochrome SCRPs but necessary to induce sizable effects at the chosen low oscillating magnetic field amplitude.^75^ Specifically, such a long lifetime was chosen so that even a single component in the broadband RF-MF could have an observable effect on the spin dynamics.^75^ Their study concluded that RF-MFs of amplitudes in the range of nanotesla do not significantly affect the time evolution of the SCRP with a realistic lifetime as long as the current knowledge of the RPM holds. Furthermore, Hiscock directly stated that an effect through an RF-MF in the context of a RPM-based magnetoreceptor will only be viable if the following points do not have to be true simultaneously:^272^

- The spin coherence time of the SCRP is extraordinarily long.
- The current model of SCRP magnetoreception is incomplete and crucial mechanisms that would increase the sensor sensitivity to weak fields are missing.
- Magnetic or electric components of the RF fields interact with the animal in some unknown way other than a direct effect on the SCRP to cause disorientation of migratory species.
- There is a systematic problem with the experimental paradigm, which introduces an artifact into all behavioral studies, and this is the origin of the observed effect.

**Figure 12:**
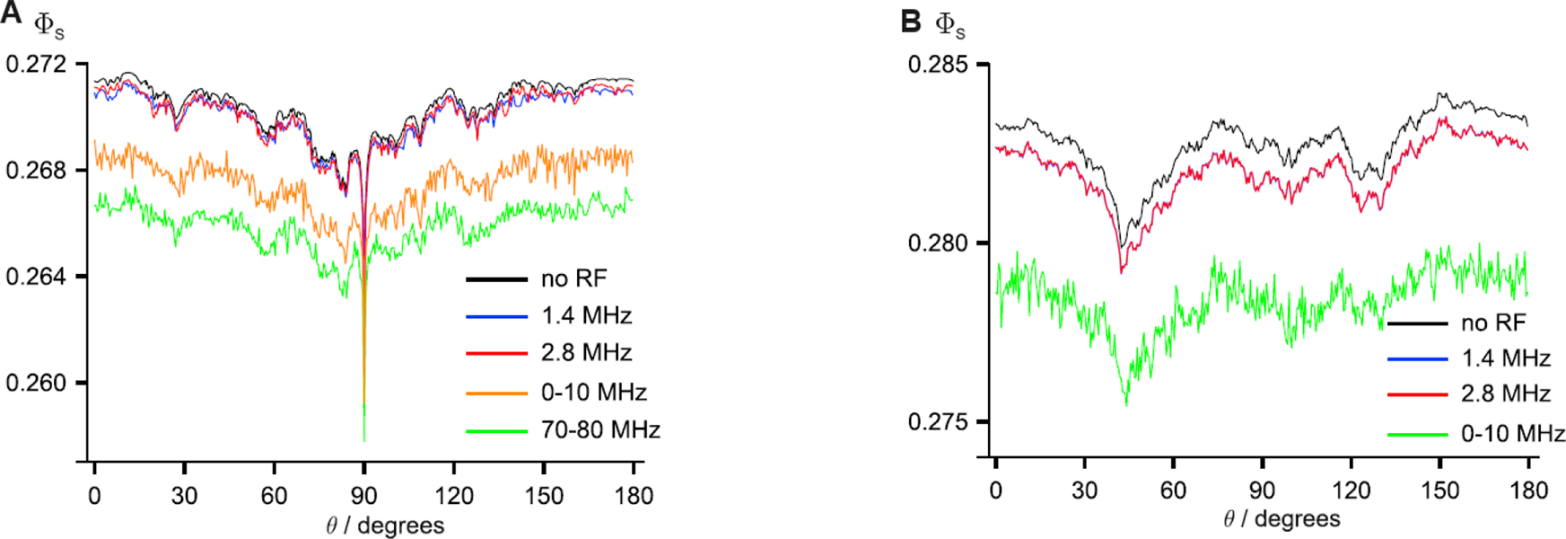
(A) Anisotropic singlet yield, Φ_S_, in the presence of 1.4 and 2.8 MHz single-frequency fields and 0–10 and 70–80 MHz broadband fields (M = $\sqrt{10^{4}}$ ) without dipolar coupling for a FAD–Trp SCRP including seven hyperfine interactions. The 1.4 MHz (blue) and 2.8 MHz (red) traces are very similar to the singlet yield in the absence of a RF-MF (black). B) The anisotropic singlet yield, Φ_*S*_, in the presence of 1.4 and 2.8 MHz single-frequency fields and a 0–10 MHz broadband field and the consideration of dipolar coupling for the FAD-Trp SCRP. The 1.4 MHz (blue) and 2.8 MHz (red) traces are almost indistinguishable. Reproduced with permission from ref 75. Copyright 2017 Biophysical Society.

On the basis of current theoretical knowledge, it appears to be exceedingly unlikely that spin coherence may be preserved for milliseconds, which would imply that the protein is expected to be severely constrained, which is implausible under normal biological conditions. As for the second point, novel advanced theories such as the radical triad,^273^ the scavenger systems,^274^ driven systems,^198^ or multiradical systems^214^ might produce new insight that provides a new understanding on how a modified RPM may interact with very weak oscillating MFs. The third statement would imply that within a precise biological system, either amplification mechanisms would exist or the current research has neglected fundamental interaction mechanisms between MFs and SCRP. The former would only be more likely for a small subset of biological systems optimized for receiving information through weak MFs. However, these optimizations would be unlikely in most of the cases reported in experiments. The last statement of Hiscock aligns with many criticisms explored in this review. It becomes apparent that all theoretical studies on the RPM and the effects of weak RF-MFs lead to similar results, revealing that a possible magnetic field effect is small with the current state of understanding the RPM and the underlying theory. However, the amount of theoretical studies on RPM-based weak RF-MF effects conducted (especially for more realistic systems) remains small, and more research is required to shed new light on possible biological effects mediated through the RPM.

## 13 Discussion

The presented experimental studies and the employed theory of the potential effects of weak RF-MF based on an RPM reveal significant discrepancies. Several experimental studies claim that biological effects through weak RF-MFs are found and associate the RPM as the underlying mechanism directly. However, the theoretical framework of spin dynamics and current knowledge often cannot reproduce these effects or at least cannot set the calculated marginal effects into a biological context. Even more drastically, the more realistic the SCRP models employed, the less likely a possible effect through an RF-MF can be noted. Furthermore, theoretical studies reveal that with the current understanding of the RPM, an extraordinarily long coherence lifetime of the SCRP^75^ and thus nearly no decoherence effect due to the environment must be present to obtain significant weak RF-MF effects.^73^ However, it is possible to present a qualitative picture of transitions using the action-histogram approach^57^ and the eigenvalue difference within an SCRP are in alignment with the experimentally observed frequency domain of RF-MF effects. Still, these calculations will not resemble the realistic spin dynamics of the SCRP under the influence of an RF-MF. The studies discussed here demonstrate that the theoretical evaluation of potential RF-MF effects within the RPM is still rarely used.

Due to the current limitations of computational methods, only a few investigations were possible, especially in the context of realistic spin systems. Further research is required to accurately describe realistic SCRPs in biological systems and to study the effects of weak RF-MFs (monochromatic and broadband) on larger spin systems that incorporate all spin interactions, which may be of a time-independent or time-dependent nature. Methods such as the SSE are promising candidates for carrying out calculations that are capable of including all important aspects of an SCRP in a biological environment.^265^ However, as demonstrated before, several parameters are used in spin dynamics calculations, which have to be obtained from electronic structure calculations or experiments. These quantities must be acquired accurately to produce reasonable results in the spin dynamics simulations. Here, multiscale approaches^275^ may be helpful tools. However, one of the most challenging parameters to obtain is the lifetime of the SCRP itself or, in other words, the incoherent processes that reduce the spin population over time through chemical reactions or electron transfer processes.

Unfortunately, this property is one of the most important in terms of discrepancies between theory and experiment. Due to the complex nature of biological systems, understanding selective reaction pathways is mandatory before further conclusions can be drawn. Current theoretical studies point in a similar direction and are still in conflict with the experiments. Nevertheless, the qualitative emergence of weak MFEs in general motivated the development of novel theoretical hypotheses, such as triad systems,^126,273^ multispin systems^214^ or the chirality-induced spin-selective (CISS) effect,^276^ which indicate promising magnetic field effects for the cryptochrome-based inclination compass and weak static magnetic fields. These theories have not yet been explored in the context of RF-MFs and the RPM and may reveal new insights into potential magnetic field effects through weak oscillating magnetic fields.

The experimental evaluation of the effects of weak RF-MFs on biological systems remains challenging. Several studies received heavy criticism because of the lack of information about dosimetry and/or the nonreproducibility. At such low field strengths and energies, executing a particular experiment several times independently becomes demanding in order to distinguish between the effects of external noise and real observable effects. Furthermore, it is crucial to combine different spectroscopic techniques for precise analysis when studying a biological system to produce greater certainty about observed effects. It becomes clear that theoretical approaches and experiments in their current state suffer from several problems. Figure 13 provides an overview of many of the challenges for experiment and theory of which one must be aware to produce meaningful results in the future (for further statements, we guide the reader to the Supporting Information of ref 139 for another discussion considering the evaluation of experimental results). As was presented in this work, a possible MFE, and more precisely an LFE on the basis of the RPM, is generally not straightforwardly achieved. Even without a time-dependent MF, reliable experimental evidence for an LFE in biological systems is quite rare. For example, in the case of cryptochrome proteins, which are studied due to their possible connection to magnetoreception, signatures of a possible LFE were observed in the lower millitesla range.^155^ However, the required experimental setup to observe such phenomena remains sensitive, e.g., to external noise, and thus, the noisiness incorporated in a biological environment will influence the detected observable significantly. Considering the difficulty in terms of a general LFE it becomes even more challenging to measure possible effects when oscillating magnetic fields with an RMS flux density of <50 μT are employed. It becomes imperative that this research field requires sophisticated statistical designs such as the round-robin test^277^ to produce reliable results.

**Figure 13:**
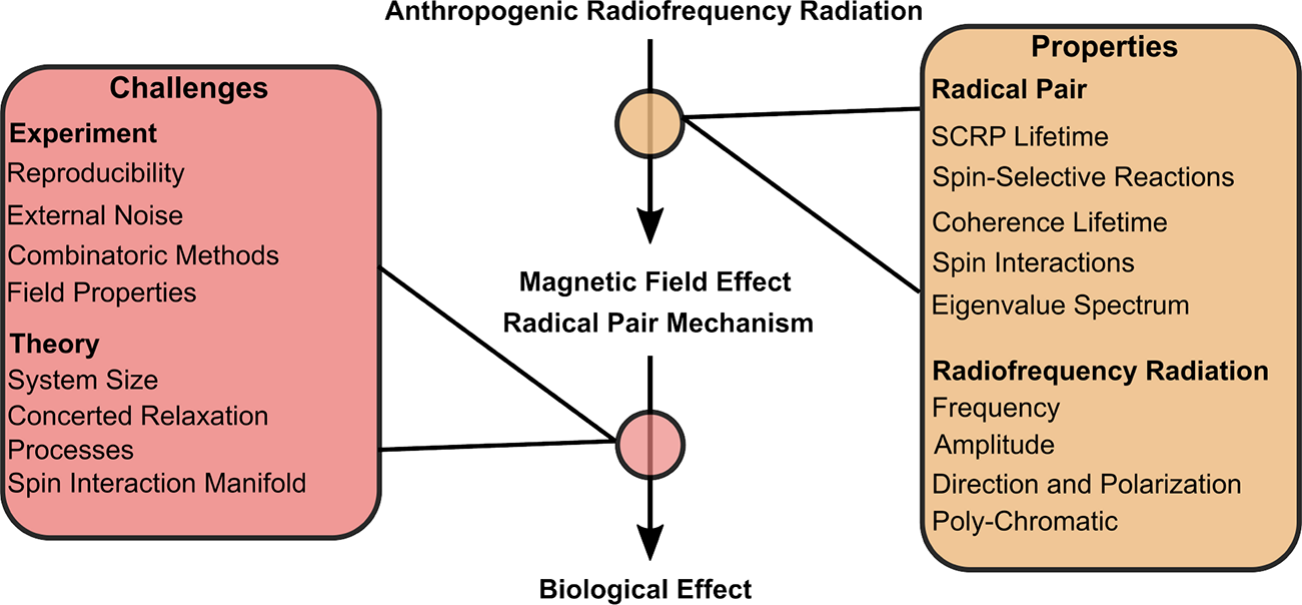
Overview of the required properties of a SCRP and RF-MFs to understand the interaction and subsequent possible effects of the RPM under the paradigm of the current knowledge (right side). If these properties are known, first prognoses can be made if an MFE might be possible. Furthermore, all current challenges for experimental and theoretical studies are listed which must be tackled when investigating a specific SCRP in a biological system.

## 14 Concluding Remarks

This review provides a detailed overview of the current state of research on weak RF-MF-induced biological effects in the context of the RPM. While several experimental studies suggest observable effects, their reproducibility and dosimetry remain significantly challenging. The RPM is frequently proposed as a mechanistic explanation, yet current theoretical models based on spin dynamics often struggle to fully account for experimental findings.

As demonstrated, accurately modeling the RPM is nontrivial, requiring consideration of multiple interactions and features. Essential interactions, such as spin–orbit coupling or dipolar coupling, are often overlooked in studies of potential MFEs. Additionally, decoherence due to spin relaxation and the short lifetimes of SCRPs impose further limitations on the detectability of weak RF-MF effects.

Despite these challenges, this review highlights a clear need for more systematic and interdisciplinary investigations. Experimental studies require robust statistical designs, standardized methodologies, and improved dosimetry to enhance reproducibility. Meanwhile, theoretical approaches must incorporate the full complexity of spin dynamics beyond simplified models. Advances such as the SSE method offer promising directions to overcome current limitations.

Ultimately, bridging the gap between experiment and theory will be key to unraveling the role of the RPM in weak RF-MF effects. Future research integrating rigorous theoretical frameworks with high-quality experimental data will be essential to clarify whether and how weak RF fields influence biological systems.

## Abbreviations

AM: amplitude modulation
BRW: Bloch–Redfield–Wangsness
DNA: deoxyribonucleic acid
ELF: extremely low frequency
EM: electromagnetic
EMF: electromagnetic field
EPR: electron paramagnetic resonance
ETC: electron transport chain
ETF: electron-transferring flavin protein
FAD: flavin adenine dinucleotide
FT: Floquet theory
GMF: geomagnetic field
LF-MF: low-frequency magnetic field
LFE: low-field effect
MC: Monte Carlo
MD: molecular dynamics
MF: magnetic field
NADPH: nicotinamide adenine dinucleotide phosphate
NZ: Nakajima–Zwanzig
RF-EMF: radiofrequency electromagnetic field
RF-MF: radiofrequency magnetic field
RFA: rotating frame approximation
RMS: root-mean square
ROS: reactive oxygen species
RP: radical pair
RPM: radical pair mechanism
SAR: specific absorption rate
SCRP: spin-correlated radical pair
SMF: static magnetic field
SSE: stochastic Schrödinger equation
Trp: tryptophan
WHO: World Health Organization
